# Loss of Caveolin-1 in Metastasis-Associated Macrophages Drives Lung Metastatic Growth through Increased Angiogenesis

**DOI:** 10.1016/j.celrep.2017.11.034

**Published:** 2017-12-05

**Authors:** Ward Celus, Giusy Di Conza, Ana Isabel Oliveira, Manuel Ehling, Bruno M. Costa, Mathias Wenes, Massimiliano Mazzone

**Affiliations:** 1 Lab of Tumor Inflammation and Angiogenesis, Center for Cancer Biology (CCB), VIB, 3000 Leuven, Belgium; 2 Lab of Tumor Inflammation and Angiogenesis, Department of Oncology, KU Leuven, 3000 Leuven, Belgium; 3 Life and Health Sciences Research Institute, School of Medicine, University of Minho, Campus de Gualtar, 4710-057 Braga, Portugal; 4 ICVS/3B’s-PT Government Associate Laboratory, Braga/Guimarães, Campus de Gualtar, 4710-057 Braga, Portugal

**Keywords:** Caveolin-1, macrophages, metastasis, angiogenesis, VEGFR1, MMP9, CSF1, VEGF-A

## Abstract

Although it is well established that tumor-associated macrophages take part in each step of cancer progression, less is known about the distinct role of the so-called metastasis-associated macrophages (MAMs) at the metastatic site. Previous studies reported that Caveolin-1 (Cav1) has both tumor-promoting and tumor-suppressive functions. However, the role of Cav1 in bone-marrow-derived cells is unknown. Here, we describe Cav1 as an anti-metastatic regulator in mouse models of lung and breast cancer pulmonary metastasis. Among all the recruited inflammatory cell populations, we show that MAMs uniquely express abundant levels of Cav1. Using clodronate depletion of macrophages, we demonstrate that macrophage Cav1 signaling is critical for metastasis and not for primary tumor growth. In particular, Cav1 inhibition does not affect MAM recruitment to the metastatic site but, in turn, favors angiogenesis. We describe a mechanism by which Cav1 in MAMs specifically restrains vascular endothelial growth factor A/vascular endothelial growth factor receptor 1 (VEGF-A/VEGFR1) signaling and its downstream effectors, matrix metallopeptidase 9 (MMP9) and colony-stimulating factor 1 (CSF1).

## Highlights

• Macrophage Cav1 signaling is critical for restraining lung metastatic growth
• Cav1 deletion in macrophages favors angiogenesis at the lung metastatic site
• Cav1 suppresses VEGF-A/VEGFR1 activity and its downstream effectors, MMP9 and CSF1

## Graphical Abstract

**Figure:**
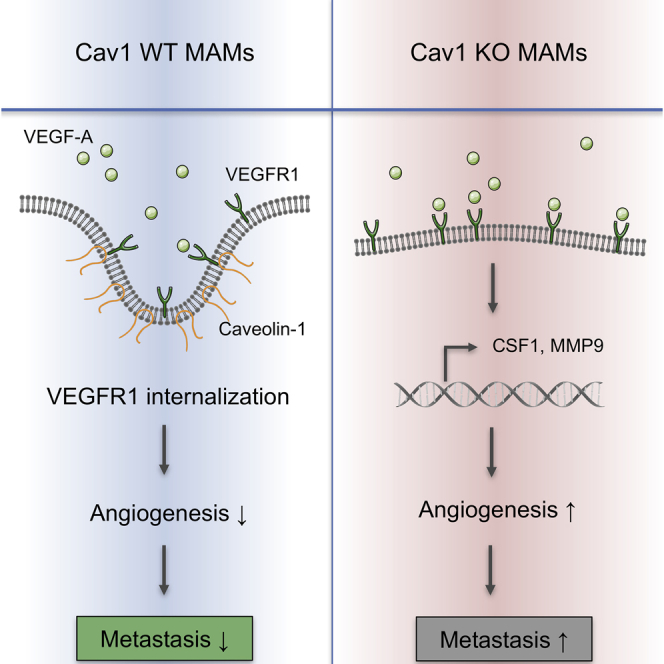

## Teaser

Celus et al. show an intrinsic anti-metastatic surveillance mechanism in the lung microenvironment whereby upregulation of Caveolin-1 in metastasis-associated macrophages specifically controls the excessive exposure of VEGFR1 at the cell surface and thereby limits downstream MMP9 and CSF1 expression, angiogenesis, and metastatic growth.

## Introduction

During cancer progression, a heterogeneous population of bone-marrow-derived cells (BMDCs) is recruited to the primary tumor site as well as to the pre-metastatic niche or established metastasis (Joyce and Pollard, 2009). Among all BMDCs, macrophages represent the most abundant population and the cells most extensively studied thus far. The plasticity of these cells has long been recognized, and it is known that macrophages carry the potential to not only sustain cancer progression and immunosuppression but also elicit cytotoxicity and antigen presentation and thus immune responses against cancer (Allavena et al., 2008). This opposite response is strictly dependent on the microenvironmental stimuli these cells are subjected to, which can vary from tumor to tumor and from the different niches they occupy (Casazza et al., 2013, Henze and Mazzone, 2016).

Although it is now well established that tumor-associated macrophages (TAMs) take part in each step of cancer growth (Ruffell and Coussens, 2015), less is known about the distinct role of so-called metastasis-associated macrophages (MAMs) at the metastatic site. Several studies have demonstrated different mechanisms through which MAMs support cancer malignancy (Hiratsuka et al., 2002, Qian et al., 2015); however, it is also possible that the immune system induces dormancy in cancer cells that have extravasated to the metastatic site and thus blocks their proliferation and growth (Sosa et al., 2014). Studies in mice using sarcoma, melanoma, and pancreatic cancer models have all pointed to CD4^+^ T cells and/or CD8^+^ cytotoxic T cells as the main executors of this metastatic dormancy (Eyles et al., 2010, Koebel et al., 2007, Müller-Hermelink et al., 2008). When looking at the involvement of innate immunity in this process, little is known about the ability of neutrophils and macrophages at the metastatic site to prevent the dissemination or growth of cancer cells (Granot et al., 2011, López-Lago et al., 2013).

Here, we describe how Caveolin-1 (Cav1) in MAMs prevents metastatic growth in the E0771 orthotopic breast cancer model, the subcutaneous Lewis lung carcinoma (LLC) model and the genetically engineered polyoma middle T (MMTV-PyMT) spontaneous breast cancer model. Cav1 is the major component of endocytic caveolae plasma membrane invaginations, and as such, it can control the turnover and activity of several proteins and signaling pathways, such as endothelin B, epidermal growth factor receptor (EGFR), insulin-like growth factor receptor 1 (IGFR-l), and transforming growth factor β (TGF-β) (Couet et al., 1997, He et al., 2015, Ravid et al., 2005, Yamaguchi et al., 2003). In general, Cav1 has been reported to have both tumor-promoting and tumor-suppressive functions, being pro- or anti-survival depending on the cancer cell type (Felicetti et al., 2009, Sunaga et al., 2004, Tanase et al., 2009, Witkiewicz et al., 2010). It is thus clear that the Janus properties of Cav1 are likely due to cell-specific effects, physiological context, and cancer stage. In addition, Cav1 has been characterized in some cell populations of the tumor stroma, where it also shows contrasting effects. For example, in tumor endothelial cells, the expression of Cav1 has been reported to modulate angiogenesis and vascular permeability both positively and negatively (Gratton et al., 2003, Lin et al., 2007, Morais et al., 2012). In cancer-associated fibroblasts, Cav1 underlines matrix stiffness and favors tumor invasion and metastasis (Goetz et al., 2011), whereas others have shown that its loss in fibroblasts correlates with poor prognosis (Capozza et al., 2012, Simpkins et al., 2012). However, the role of Cav1 in BMDCs, particularly macrophages, was never disclosed.

Using chimeric mice, which are Cav1 wild-type (WT) or knockout (KO) in the bone marrow compartment, we describe here a mechanism by which Cav1 in MAMs restrains vascular endothelial growth factor A/vascular endothelial growth factor receptor 1 (VEGF-A/VEGFR1) signaling and its downstream effectors, matrix metallopeptidase 9 (MMP9) and colony-stimulating factor 1 (CSF1). Gene targeting of Cav1 in MAMs unleashes VEGFR1^+^ MAMs and MMP9 activity, which in turn favors the angiogenic switch and metastatic growth. With this in mind, we define here an anti-metastatic function of Cav1 in macrophages.

## Results

### Deletion of Cav1 in BMDCs Leads to Increased Metastasis in Multiple Tumor Models

**Figure 1:**
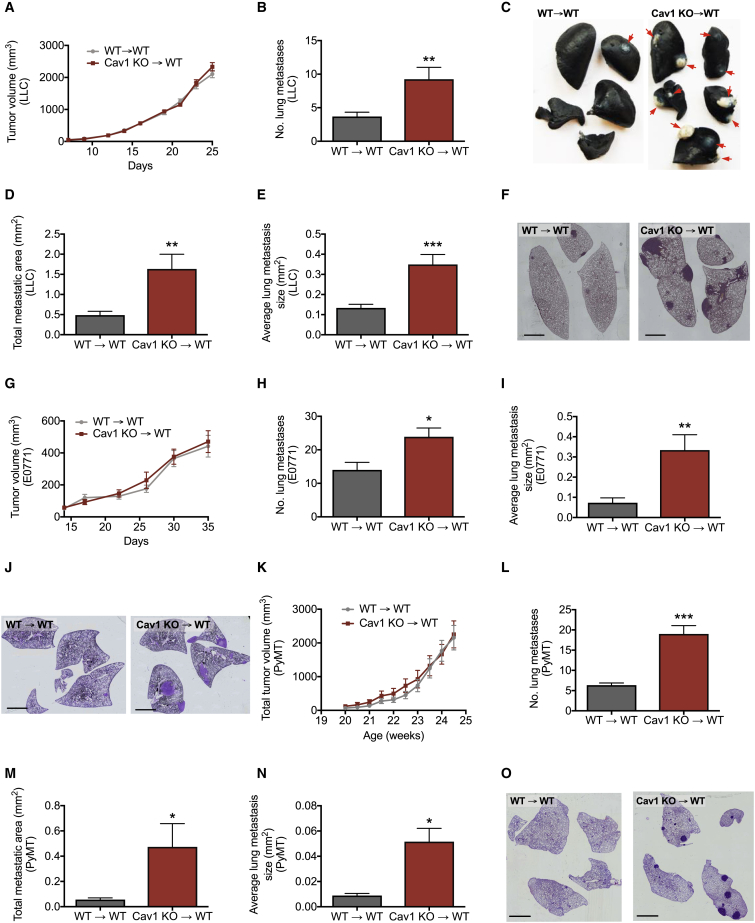
Deletion of Cav1 in BMDCs Leads to Increased Metastasis in Multiple Tumor Models (A–F) LLC tumor growth (A) of lethally irradiated WT mice reconstituted with WT (WT→WT) or Cav1 KO (Cav1 KO→WT) BM cells. Quantification of the number of lung metastatic nodules (B) and representative images (C). Metastatic nodules are indicated by arrows. Histological analysis of lung metastasis showing the total metastatic area per mouse (D), average size of each metastasis (E), and representative mosaic images of H&E stained lung sections (F). Scale bar, 2 mm. n = 30 mice per genotype. (G–J) Tumor growth (G) and number of lung metastatic nodules (H) in an orthotopic E0771 breast cancer model. Histological analysis of lung metastasis showing the average size of each metastasis (I) and representative mosaic images of H&E stained lung sections (J). Scale bar, 2 mm. n = 8 mice per genotype. (K–O) Total tumor growth (K) and number of lung metastatic nodules (L) of lethally irradiated PyMT mice reconstituted with WT or Cav1 KO BM cells. Histological analysis of lung metastasis showing the total metastatic area per mouse (M), the average size of each metastasis (N), and representative mosaic images of H&E stained lung sections (O). Scale bar, 2 mm. n = 7 mice per genotype. ^∗^p < 0.05, ^∗∗^p < 0.01, and ^∗∗∗^p < 0.001 versus WT→WT. All graphs show mean ± SEM.

In order to investigate the role of Cav1 in BMDCs, we transplanted bone marrow (BM) from WT or *Cav1* KO mice into lethally irradiated WT recipient mice, WT→WT and Cav1 KO→WT, respectively. Upon reconstitution, Cav1 KO→WT chimeras displayed normal blood counts comparable to those of WT→WT mice (Table S1). We then implanted LLC cells subcutaneously. Although primary tumor growth and weight were not different between both groups of chimeric mice (Figures 1A and S1A), Cav1 KO→WT chimeras had 2-fold more lung metastases than WT→WT chimeras (Figures 1B and 1C). Histologically, the total metastatic area was increased upon bone marrow Cav1 deletion, as well as the average size of individual lung metastasis (Figures 1D–1F).

In an alternative tumor model, which was obtained by injecting E0771 breast cancer cells orthotopically in the mammary fat pad, lung metastases were significantly increased upon deletion of Cav1 in the BM, whereas primary tumor growth was similar between groups (Figures 1G, 1H, and S1B). Histological quantifications showed a drastic increase in the average size of individual lung metastasis in Cav1 KO→WT chimeras as compared to WT→WT mice (Figures 1I and 1J). In order to further confirm the anti-metastatic role of Cav1 in BMDCs, we used the polyoma middle T (MMTV-PyMT) spontaneous breast cancer mouse model, which resembles the characteristics of human luminal breast cancer (Lin et al., 2003). Consistent with our previous models, we transplanted the BM from WT or Cav1 KO mice in 12-week-old lethally irradiated PyMT recipient mice (8 weeks before the first tumor onset). We observed that BM *Cav1* deletion in these mice did not affect PyMT primary tumor growth or weight (Figures 1K and S1C). However, the number of metastatic nodules in the lung was much higher in Cav1 KO→WT PyMT chimeras than in WT→WT PyMT controls (Figure 1L). Histological quantifications showed that the total metastatic area, as well as the average size of individual lung metastases, was also increased in PyMT mice upon BM *Cav1* deletion (Figures 1M–1O). Together, these data suggest that Cav1 in BMDCs has an anti-metastatic function in the lungs without affecting primary tumor growth at other sites.

### Macrophages Are Responsible for Increased Metastasis in Cav1 KO→WT Chimeras

**Figure 2:**
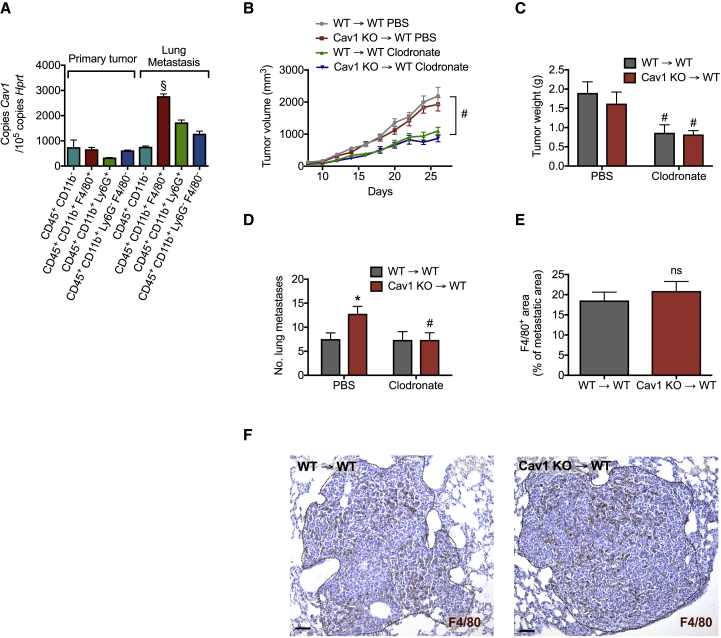
Macrophages Are Responsible for Increased Metastasis in Cav1 KO→WT Chimeras (A) *Cav1* expression in CD45^+^CD11b^−^ non-myeloid cells, CD45^+^CD11b^+^F4/80^+^ macrophages (TAMs), CD45^+^CD11b^+^Ly6G^+^ neutrophils, and other CD45^+^CD11b^+^Ly6G^−^F4/80^−^ myeloid cells derived from the primary tumor and CD45^+^CD11b^−^ non-myeloid cells, CD45^+^CD11b^+^F4/80^+^ macrophages (MAMs), CD45^+^CD11b^+^Ly6G^+^ neutrophils, and other CD45^+^CD11b^+^Ly6G^−^F4/80^−^ myeloid cells sorted from metastatic nodules of LLC-tumor-bearing mice. n = 4 mice. (B–D) Tumor growth (B), weight (C), and number of lung metastatic nodules (D) in LLC-tumor-bearing mice treated with macrophage-depleting clodronate liposomes or PBS. n = 11 mice per genotype. (E and F) F4/80^+^ macrophage accumulation in LLC metastasis (E) and representative images (F). Scale bar, 100 μm. n = 8 mice per genotype. ^§^p < 0.05 versus other cell populations, ^∗^p < 0.05 versus WT→WT, and ^#^p < 0.05 versus PBS. ns, not significant. All graphs show mean ± SEM.

In tumor-bearing mice, MAMs isolated from lung metastases displayed the highest levels of *Cav1* expression in comparison to TAMs or other immune cells sorted from either the primary tumor or pulmonary metastasis (Figure 2A). Prompted by these data, we evaluated whether Cav1 in macrophages played a role during metastatic growth. Using clodronate liposomes, we achieved an ∼55%–60% reduction in macrophage infiltration at the primary tumor and metastatic sites (Figure S2). In this setting, the difference in metastasis between WT→WT and Cav1 KO→WT chimeras was completely abrogated (Figure 2D). At the level of the primary site, clodronate-liposome treatment caused equal tumor growth and weight reduction in both WT→WT and Cav1 KO→WT chimeras (Figures 2B and 2C). However, both TAM and MAM infiltration was not affected by *Cav1* deficiency (Figures 2E, 2F, S2A, and S2B). Overall, these data suggest that Cav1 deletion in macrophages is responsible for increased metastasis.

### The Pro-metastatic Function of Cav1 KO Macrophages Is Likely Restricted to the Lungs

**Figure 3:**
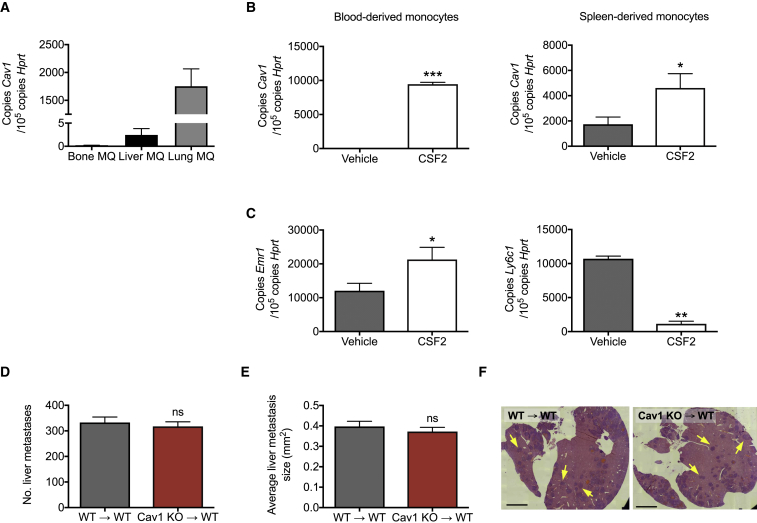
The Pro-metastatic Function of Cav1 KO Macrophages Is Likely Restricted to the Lungs (A) *Cav1* expression in CD45^+^CD11b^+^F4/80^+^ macrophages (MQ) sorted from bone, liver, and lung tissue of healthy mice. n = 10 mice. (B and C) *Cav1* expression in blood- and spleen-derived monocytes treated for 7 days with GM-CSF (CSF2) or vehicle (B), and expression of *Ly6C1* and *Emr1* at day 7 in spleen-derived monocytes (C). n = 4 mice. (D–F) Number of liver metastatic nodules upon portal vein injection of E0771 cells (D). Histological analysis of hepatic metastasis showing the average size of each metastasis (E) and representative mosaic images of H&E stained liver sections (F). Metastatic nodules are indicated by arrows. Scale bar, 2 mm. n = 5 mice per genotype. ^∗^p < 0.05, ^∗∗^p < 0.01, and ^∗∗∗^p < 0.001 versus vehicle. ns, not significant. All graphs show mean ± SEM.

Interestingly, when measuring the expression of Cav1 in different tissues of healthy mice, we found that only CD45^+^CD11b^+^F4/80^+^ interstitial macrophages in the lungs, but not hepatic or bone-marrow-resident macrophages, displayed abundant *Cav1* expression levels (Figure 3A). Fu et al. (2012) previously showed that monocytes treated for 7 days with CSF2 (also known as granulocyte-macrophage colony-stimulating factor [GM-CSF]) displayed increased expression levels of *Cav1*. Consistent with these findings, we observed that 7 days of CSF2 treatment could induce high *Cav1* expression levels in both blood- and spleen-derived Ly-6C^+^F4/80^low/−^Ly6G^−^ monocytes (Figure 3B). Downregulation of *Ly6c1* and upregulation of *Erm1* expression upon treatment with CSF2 supports the differentiation of monocytes into macrophages (Figure 3C). These data suggest that upon monocyte arrival to the lungs and during their transition into macrophages, *Cav1* expression can be induced by CSF2, a factor known to play a key role in the homeostatic control of the pulmonary macrophage phenotype (Kopf et al., 2015, Lavin et al., 2015, Mantovani and Allavena, 2015). Based on these observations, we then tested whether the organ context plays a role in the observed phenotype. For this, we examined breast cancer metastasis to the liver by performing portal vein injection of E0771 breast cancer cells (Goddard et al., 2016). After 10 days, we detected no difference in the number of metastatic nodules in the liver between Cav1 KO→WT chimeras and WT→WT mice (Figures 3D–3F). Altogether, we conclude that the effects of *Cav1* deletion in MAMs are likely restricted to the lung parenchyma.

### Cav1 Deletion in MAMs Leads to Increased Metastatic Growth and Angiogenesis

**Figure 4:**
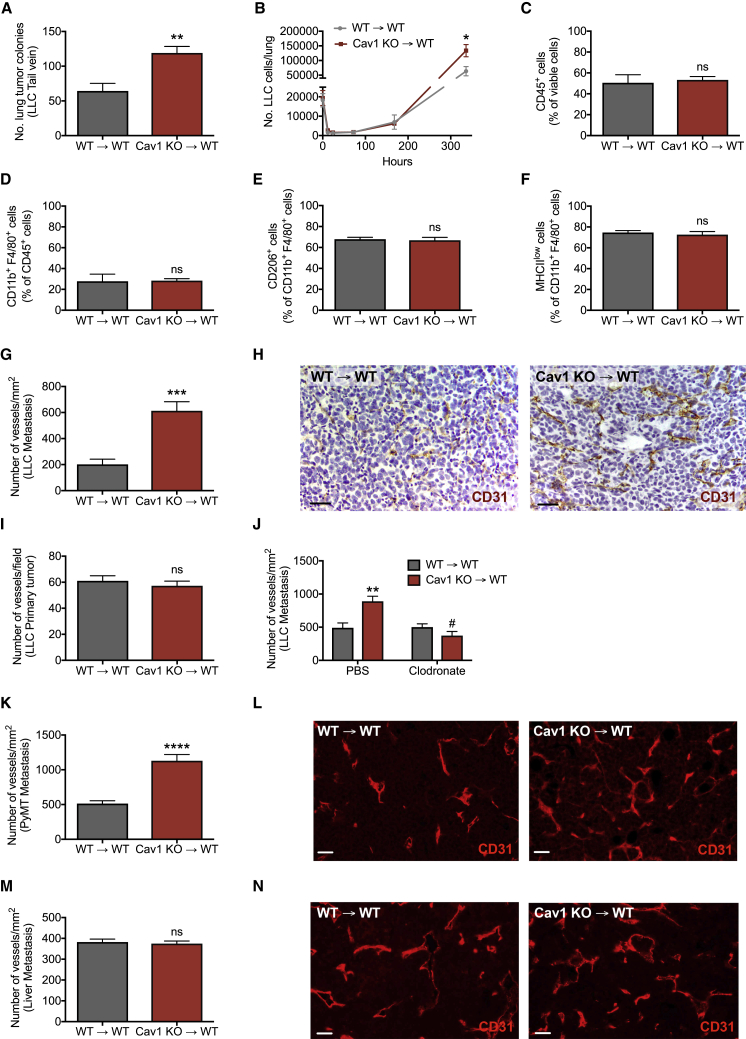
Cav1 Deletion in MAMs Leads to Increased Metastatic Growth and Angiogenesis (A) Number of lung metastatic nodules upon tail vein injection of LLC cells. Total mice, n = 6 per genotype. (B) Tumor cell extravasation measured by qRT-PCR analysis on whole lungs upon tail vein injection of LLC cells. Total mice, n = 4 per condition. (C–F) FACS quantification of total CD45^+^ leukocytes (C), F4/80^+^ MAMs (D), M2-like CD206^+^ MAMs (E), and MHC class II^low^ MAMs (F) in WT→WT and Cav1 KO→WT lung metastatic lesions. n = 4 mice per genotype. (G–I) Number of CD31^+^ blood vessels in LLC metastatic lesions (G) and representative images (H). Scale bar, 50 μm. Number of CD31^+^ blood vessels in primary LLC tumors (I). n = 10 mice per genotype. (J) Number of CD31^+^ blood vessels in LLC metastatic lesions upon treatment with clodronate liposomes or PBS. n = 9 mice per condition. (K and L) Number of CD31^+^ blood vessels in lung metastatic lesions of PyMT mice (K) and representative images (L). Scale bar, 50 μm. Total mice, n = 7 per genotype. (M and N) Number of CD31^+^ blood vessels in E0771 metastatic lesions in the liver (M) and representative images (N). Scale bar, 50 μm. n = 5 mice per genotype. ^∗∗^p < 0.01, ^∗∗∗^p < 0.001, and ^∗∗∗∗^p < 0.0001 versus WT→WT. ^#^p < 0.05 versus PBS. ns, not significant. All graphs show mean ± SEM.

Because we observed that MAMs show much higher *Cav1* expression than TAMs (Figure 2A), we hypothesized that the effect of Cav1 on metastasis is independent of the primary tumor. We therefore injected LLC cells directly into the bloodstream. After 20 days, we found a significantly higher number of pulmonary metastatic nodules in Cav1 KO→WT versus WT→WT mice (Figure 4A). While *Cav1* deletion in KO→WT chimeras positively affected metastatic cancer cell growth at a late stage, their extravasation and lodging in the early stage after intravenous cancer cell injection was not affected (Figure 4B). We can therefore argue that Cav1 KO macrophages represent supportive MAMs, which mediate the escape from tumor cell dormancy and increase tumor cell proliferation independently of the primary tumor and initial tumor cell extravasation.

In an attempt to further characterize the phenotype of Cav1 KO macrophages in the metastatic niche, we evaluated features of the MAM infiltrate by fluorescence-activated cell sorting (FACS) and analyzed the expression of the M1-like marker major histocompatibility complex class II (MHC class II) and the M2-like marker CD206 (Mrc1) on the cell membrane of F4/80^+^ cells (Laoui et al., 2014). The abundance of immune cells in general and specifically of macrophages in the metastasis was comparable in both groups of chimeric mice (Figures 4C and 4D). Additionally, Cav1 KO→WT chimeras displayed similar numbers of MHC class II^low^ and CD206^high^ MAMs as compared to WT→WT controls (Figures 4E and 4F). Additionally, Cav1 KO→WT chimeras had numbers of metastasis-infiltrating CD4^+^ and CD8^+^ T cells (TCR-β^+^ cells), neutrophils (CD11b^+^Ly6G^+^ cells), natural killer (NK) cells (NKp46^+^ cells), and B cells (CD45R^+^ cells) similar to WT→WT mice (Figure S3). Gene expression markers typically used to characterize the classically (M1-like) and alternatively activated (M2-like) macrophage phenotype were also unaltered in MAMs derived from WT→WT and Cav1 KO→WT chimeras (Figure S4), suggesting that deletion of Cav1 does not affect macrophage polarization.

During our attempt to understand the biological reasons underlying the metastatic boost upon hematopoietic deletion of Cav1, we found that vessel density in the metastatic lesion was markedly augmented in Cav1 KO→WT versus WT→WT chimeras (Figures 4G and 4H). However, blood vessel density in LLC primary tumors was comparable in both groups of chimeric mice (Figure 4I). The enhanced angiogenesis at the metastatic site in Cav1 KO→WT mice was completely prevented by clodronate-liposome treatment (Figure 4J). Importantly, the observed angiogenic phenotype was consistent in our spontaneous breast cancer model and was organ specific, because PyMT lung metastases in Cav1 KO→WT chimeric mice showed increased blood vessel density, whereas experimental liver metastases did not (Figures 4K–4N).

We also studied the functionality and vascular integrity of the blood vessels in the pulmonary metastases. However, blood vessels in Cav1 KO→WT metastatic lesions did not show any differences in normalization and permeability as compared to WT→WT mice in terms of hypoxic area, pericyte coverage, and number of leaked red blood cells in the perivascular space (Figures S5A–S5C). In addition, the perivascular localization of macrophages in the pulmonary metastatic lesions did not display any significant differences between WT→WT and Cav1 KO→WT mice (Figure S5D). Altogether, these data suggest that MAM-associated Cav1 negatively regulates the sprouting of new blood vessels (but not their function), and this occurs specifically at the lung metastatic site, where Cav1 is abundantly expressed by interstitial macrophages.

### Augmented VEGFR1 Activity upon Cav1 Deletion in Macrophages Leads to Increased MMP9-Mediated Metastatic Growth and Angiogenesis

**Figure 5:**
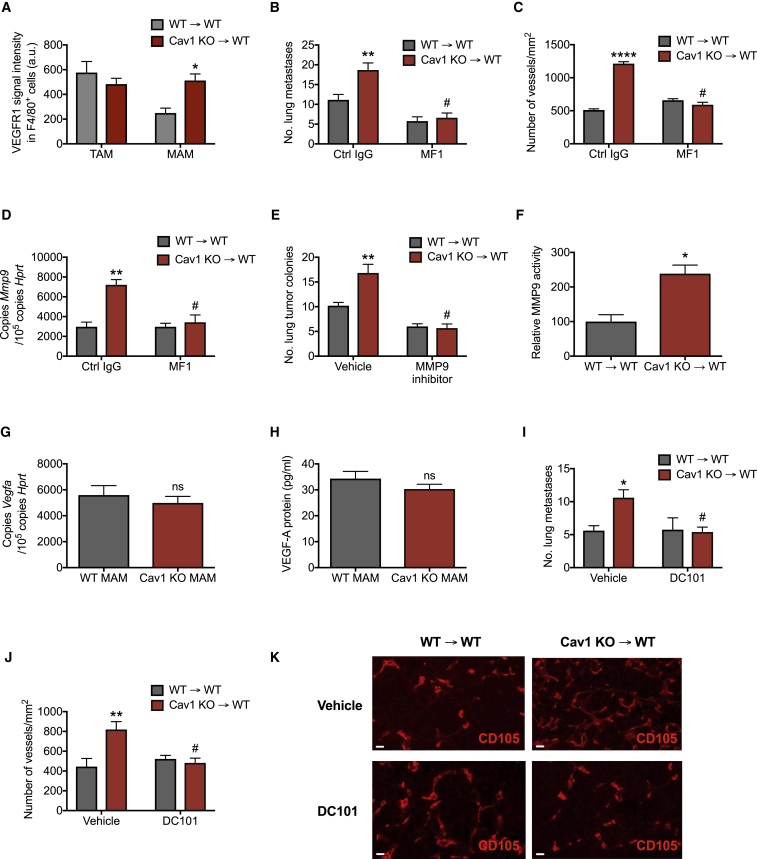
Augmented VEGFR1 Activity upon Cav1 Deletion in Macrophages Leads to Increased MMP9-Mediated Metastatic Growth and Angiogenesis (A) FACS analysis of the mean VEGFR1 signal intensity on CD45^+^CD11b^+^F4/80^+^ macrophages in LLC primary tumors (TAMs) or metastatic lesions (MAMs). n = 4 mice per genotype. (B–D) Number of lung metastatic nodules upon tail vein injection of LLC cells in mice treated with VEGFR1-blocking antibodies (MF1) or control IgG (Ctrl IgG) (B), and number of CD105^+^ blood vessels in metastatic lesions (C). *Mmp9* gene expression in FACS-sorted CD45^+^CD11b^+^F4/80^+^ MAMs from mice treated with VEGFR1-blocking antibodies (MF1) or control IgG (Ctrl IgG) (D). Total mice, n = 5 per genotype. (E) Number of lung metastatic nodules upon tail vein injection of LLC cells in mice treated with MMP9 inhibitor or vehicle. n = 7 mice per condition. (F) MMP9 activity in FACS-sorted CD45^+^CD11b^+^F4/80^+^ MAMs measured by gelatin zymography. n = 5 mice per genotype. (G and H) *Vegfa* gene expression in FACS-sorted CD45^+^CD11b^+^F4/80^+^ MAMs from WT→WT and Cav1 KO→WT chimeras (G). Protein levels of VEGF-A, measured by ELISA, in the cell supernatants of FACS-sorted CD45^+^CD11b^+^F4/80^+^ MAMs from WT→WT and Cav1 KO→WT chimeras (H). n = 4 mice per genotype. (I–K) Number of lung metastatic nodules upon tail vein injection of LLC cells in mice treated with VEGFR2-blocking antibodies (DC101) or vehicle (I), number of CD105^+^ blood vessels in lung metastatic lesions (J), and representative images (K). Scale bar, 50 μm. n = 6 mice per condition. ^∗^p < 0.05, ^∗∗^p < 0.01, and ^∗∗∗∗^p < 0.0001 versus WT→WT. ^#^p < 0.05 versus Ctrl IgG or vehicle. ns, not significant. All graphs show mean ± SEM.

Caveolae are important structural elements involved in modulating signal transduction of several receptors such as VEGFR1, CCR2, or others depending on the cell context (Casalou et al., 2011, Catusse et al., 2009, Dzenko et al., 2001, Fu et al., 2012, Ge and Pachter, 2004). In order to elucidate the mechanism by which *Cav1* deletion leads to increased blood vessel formation, we investigated which cell surface receptors on MAMs are modulated by Cav1. Interestingly, upon *Cav1* deletion, macrophages sorted from metastatic nodules showed increased protein expression of VEGFR1 (also known as Flt1) at the membrane surface (Figure 5A), whereas *Vegfr1* transcripts were the same in both WT and Cav1 KO MAMs (Figure S6A). The upregulation of VEGFR1 in Cav1 KO MAMs was specific, because other cell surface receptors were unaltered (Figures S6B–S6D). Augmented VEGFR1 expression was found only in MAMs, because protein levels of VEGFR1 were similar in WT→WT and Cav1 KO→WT TAMs (Figure 5A). Moreover, we showed that VEGFR1 modulation by Cav1 was responsible for the observed phenotype, because treating mice with the VEGFR1-blocking antibody MF1 abrogated the increased metastatic growth and vessel formation in Cav1 KO→WT chimeras intravenously injected with LLC cells (Figures 5B and 5C). Previous studies have shown that VEGFR1 activity drives *Mmp9* expression in the context of cancer (Bergers et al., 2000, Hiratsuka et al., 2002, Li et al., 2015). Accordingly, we observed a strong increase in *Mmp9* expression in macrophages upon *Cav1* deletion, which was inhibited after MF1 treatment (Figure 5D). In addition, by using an inhibitor of MMP9, we rescued metastatic growth in Cav1 KO→WT mice down to the same levels observed in WT→WT mice (Figure 5E). Accompanying the expression data on *Mmp9*, we observed increased MMP9 activity in sorted MAMs upon deletion of Cav1 (Figure 5F).

Because VEGF-A is the most prominent angiogenic factor secreted by MAMs that stimulates vascular permeability and tumor cell extravasation, we tested the relevance of this ligand in Cav1 KO macrophages (Qian et al., 2011a). We showed that both *Vegfa* expression and secretion of VEGF-A in MAMs were unaffected upon deletion of Cav1 (Figures 5G and 5H). To rule out the possibility that other MMP9-mediated capabilities besides angiogenesis are required for the increased formation of metastasis in Cav1 KO→WT mice, we blocked angiogenesis by injecting DC101 antibodies that, by recognizing VEGFR2, specifically target endothelial cells, but not macrophages (Koch and Claesson-Welsh, 2012). At day 20 upon LLC tail vein injection, DC101 treatment in Cav1 KO→WT chimeras abrogated the increased metastatic growth and angiogenesis to the same levels as control-treated WT→WT mice (Figures 5I–5K), confirming that vessel formation is responsible for the observed phenotype.

### Enhanced VEGF-A/VEGFR1 Signaling upon Cav1 Deletion in MAMs Leads to Downstream Activation of the CSF1/MMP9 Axis

**Figure 6:**
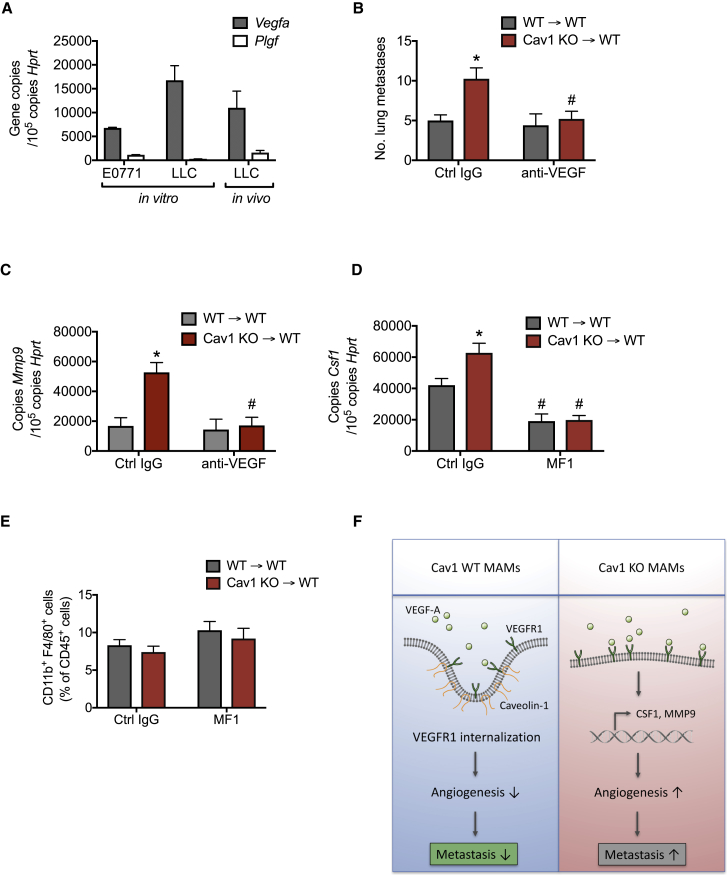
Enhanced VEGF-A/VEGFR1 Signaling upon Cav1 Deletion in MAMs Leads to Downstream Activation of the CSF1/MMP9 Axis (A) *Vegfa* and *Plgf* expression in E0771 and LLC cancer cells in culture (*in vitro*), and expression of these genes in LLC-derived metastatic lesions (*in vivo*). n = 4 mice. (B and C) Number of lung metastatic nodules upon tail vein injection of LLC cells in mice treated with anti-VEGF-A antibodies (anti-VEGF) or control IgG (Ctrl IgG) (B) and *Mmp9* gene expression in FACS-sorted CD45^+^CD11b^+^F4/80^+^ MAMs (C). n = 4 mice per condition. (D and E) *Csf1* gene expression in FACS-sorted CD45^+^CD11b^+^F4/80^+^ MAMs from mice treated with VEGFR1-blocking antibodies (MF1) or control IgG (Ctrl IgG) (D), and FACS quantification of total F4/80^+^ MAMs (E). n = 5 mice per condition. (F) Schematic overview of the data showing that deletion of Caveolin-1 (Cav1) in metastasis-associated macrophages (MAMs) drives VEGF-A/VEGFR1 activity and its downstream effectors, MMP9 and CSF1, which in turn favors the angiogenic switch and metastatic growth. Wild-type (WT) MAMs control the excessive exposure of VEGFR1 at the membrane surface via Cav1-mediated internalization, leading to a decrease in the levels of *Mmp9* and *Csf1* transcripts, which results in inhibition of metastatic growth and angiogenesis. ^∗^p < 0.05 versus WT→WT. ^#^p < 0.05 versus Ctrl IgG. All graphs show mean ± SEM.

VEGF-A and placental growth factor (PlGF) are the two VEGFR1 ligands involved in angiogenesis (Fischer et al., 2008). Of these two ligands, we found that LLC and E0771 cancer cells express high levels of *Vegfa* but barely detectable levels of *Plgf* (Figure 6A). Similar results were obtained when quantifying the amount of *Vegfa* and *Plgf* in LLC-derived lung lesions in WT mice (Figure 6A). Therefore, we decided to block only the VEGF-A ligand and assess how this affects the expression of MMP9. For this approach, WT→WT and Cav1 KO→WT mice were consecutively treated with VEGF-A-blocking antibodies at day 16 and 18 upon LLC tail vein injection. Anti-VEGF-A antibodies were able to rescue metastatic growth in Cav1 KO→ WT mice to the same levels observed in WT→WT mice (Figure 6B). Moreover, *Mmp9* expression in Cav1 KO MAMs treated with anti-VEGF-A decreased to the same levels observed in WT MAMs (Figure 6C), suggesting that VEGFR1-MMP9 signaling upon *Cav1* deletion in MAMs is mainly driven by the VEGF-A ligand.

Qian et al. (2015) have previously shown that CSF1 (also known as macrophage colony-stimulating factor [M-CSF]) is a downstream gene target of VEGFR1 in MAMs. We therefore tested the expression of CSF1 in Cav1 KO MAMs and found that Cav1 KO MAMs display significantly higher *Csf1* expression levels than WT MAMs (Figure 6D). Moreover, MF1 treatment leads to a strong abrogation of *Csf1* expression levels in Cav1 KO MAMs (Figure 6D), suggesting a possible involvement of CSF1 in the Cav1 KO macrophage phenotype. Furthermore, Qian et al. (2015) also showed that macrophage recruitment to the lung was unaffected by MF1 inhibition. We further demonstrated that MAM recruitment was indeed unaffected in either immunoglobulin G (IgG)-treated and MF1-treated Cav1 KO→WT chimeras (Figure 6E).

In conclusion, these data argue that increased VEGF-A/VEGFR1 activity upon *Cav1* deletion in MAMs drives the downstream expression of MMP9 and CSF1, altogether facilitating blood vessel formation and metastatic growth in the lungs (Figure 6F).

## Discussion

Although many studies have thoroughly characterized the role of TAMs and their plastic phenotypes at the primary tumor, far less is known about MAMs in the metastatic niche. Previous studies have shown that circulating monocytes are recruited to the lung metastatic niche and release VEGF that permeabilizes blood vessels, thus enforcing breast cancer cell extravasation (Qian et al., 2011a). Once in the lung parenchyma, metastatic breast cancer cells receive survival signals from MAMs through the interaction of vascular cell adhesion molecule-1 (VCAM-1) and α4-integrins (Chen et al., 2011). Moreover, macrophage VEGFR1 expression, while dispensable for MAM recruitment, is determinant for the regulation of a set of prometastatic genes in MAMs, such as *Csf1* in breast cancer lung metastases (Qian et al., 2015) or *Mmp9* in B16 or LLC lung metastases (Hiratsuka et al., 2002, Kaplan et al., 2005).

In contrast to these metastasis-promoting activities, our data highlight an intrinsic anti-metastatic surveillance mechanism whereby upregulation of Cav1 in MAMs controls excessive expression of VEGFR1, thus limiting expression of MMP9 and CSF1, angiogenesis, and metastatic growth (Figure 6F). These effects are limited to the metastatic niche and were not observed in the primary tumor, possibly because of two reasons. First, MAMs show a much higher expression of Cav1 than TAMs. This might be induced by the lung microenvironment, in which high *Cav1* expression is important to orchestrate appropriate innate immune reactions to pathogens and allergens (Jin et al., 2011). Second, persistent hypoxia (present in the primary tumor) is known to strongly upregulate VEGFR1 expression (Eubank et al., 2011, Okuyama et al., 2006, Roda et al., 2011), thereby possibly saturating any modulatory effects by low *Cav1* expression levels in TAMs. Indeed, hypoxic tumor areas are characterized by the presence of VEGFR1^high^ macrophages that actively secrete MMP9 and thus promote angiogenesis and invasion (Du et al., 2008). Based on our data, we suggest a model in which a VEGFR1-MMP9 signaling axis in lung MAMs is rapidly quenched by high *Cav1* expression, because the treatment of metastasis-bearing mice with the anti-VEGFR1 antibody MF1 did not result in a reduction of *Mmp9* gene expression. On the other hand, KO of Cav1 increased the membrane exposure of VEGFR1 on MAMs, resulting in increased transcription and activity of MMP9, overall inducing excessive blood vessel formation and expanded metastatic size. Nevertheless, a further decrease in lung metastases in WT→WT mice upon MMP9 inhibitor treatment was also observed, which can be explained by the inhibition of MMP9 derived from other cell types, such as endothelial cells (Hiratsuka et al., 2002).

Recent data published by Qian et al. (2015) has demonstrated that MAMs uniquely express VEGFR1 at the cell surface, which is critical for spontaneous lung metastasis, via downstream regulation of a set of inflammatory genes. Among these genes, *Csf1*, a key factor for macrophage survival, was shown to be the main target gene, promoting metastatic growth once cancer cells seed into the lungs. In an effort to link this closely related study with our observed phenotype, we discovered that Cav1 KO MAMs display higher expression levels of *Csf1* than WT MAMs, which were abrogated upon MF1 treatment. These data could indicate that *Cav1* deletion in MAMs could boost the CSF1 inflammatory cascade through excessive exposure of VEGFR1 at the cell surface. Previous studies have shown that blockage of VEGFR1 and CSF1 signaling leads to inhibition of MMP9-mediated actions and consequently suppression of metastatic growth (Kaplan et al., 2005, Priceman et al., 2010). However, further studies are warranted to unravel whether MMP9 and CSF1 act either as two epistatic processes or as one cascade where *Mmp9* expression is downstream CSF1 signaling. When seeking the VEGFR1 ligands responsible for this cascade, we found that VEGF-A, but not placental growth factor (PIGF), was expressed both *in vivo* and *in vitro*, at least in our tumor models. Together with this finding, the use of VEGF-A-blocking antibodies supports the idea that VEGFR1-MMP9 signaling upon *Cav1* deletion in MAMs is mainly driven by VEGF-A.

In the attempt to further characterize the mechanism of Cav1 signaling, we observed that monocytes, treated for 7 days with CSF2 (GM-CSF), display significantly higher *Cav1* expression levels when differentiated into mature macrophages. These data suggest that CSF2 can induce high *Cav1* expression levels in monocyte-derived macrophage subsets, such as interstitial macrophages in the lung. In this context, we can hypothesize that CSF2 might block VEGFR1-CSF1 signaling in a Cav1-dependent manner and thus suppress MMP9-CSF1 pro-metastatic signaling. These data are in agreement with previously proposed competition models between the actions of CSF1 and CSF2 (Fleetwood et al., 2007, Hamilton, 2008). However, further *in vivo* experiments are required to support the validity of this concept.

Another important aspect of this study relates to the organ specificity of the observed phenotype, meaning that the effects of *Cav1* deletion in MAMs are limited to the lung parenchyma as compared to other metastatic sites, such as the liver. We showed that, upon portal vein injection of E0771 breast cancer cells, metastatic growth and vessel density of liver metastatic lesions was unaffected upon BM deletion of Cav1. In addition, the use of subcutaneous LLCs, orthotopic E0771, and transgenic PyMT breast tumors, which all developed larger spontaneous lung metastasis in Cav1 KO→WT mice, allows us to further speculate on the results obtained upon intravenous injection of LLC cells, which also showed enhanced metastatic growth in Cav1 KO→WT mice. Because the latter is a model of lung cancer cells colonizing the pulmonary tissue, previous work has referred to this approach as an orthotopic model of primary lung tumor growth (Goto et al., 2002, Moncho-Amor et al., 2011, Ottewell et al., 2006). Therefore, it is licit to think that Cav1 in macrophages might play a suppressive role during both primary lung carcinogenesis and lung metastasis. However, *in vivo* models of lung carcinogenesis are required to validate this hypothesis.

In conclusion, the role of Cav1 has been extensively studied in cancer, with a focus on cancer cells, endothelial cells, and fibroblasts, leading to the suggestion that the Caveolin scaffolding domain can be used in the treatment of cancer (Gratton et al., 2003, Simpkins et al., 2012, Williams et al., 2004). The therapeutic administration of Cav1 scaffold protein might be a worthwhile pursuit, because the loss of Cav1 in the tumor stroma has been previously associated with more aggressive disease and poor patient outcomes in breast cancer, pancreatic cancer, and melanoma (Jia et al., 2014, Qian et al., 2011b, Wu et al., 2011). We complemented these observations by studying the role of Cav1 in immune cells and thus disclosed an anti-metastatic protective function of Cav1 that is exclusive to MAMs, but not other immune compartments.

## Experimental Procedures

More detailed methods can be found in Supplemental Experimental Procedures.

### Animals

Cav1 KO mice on a C57BL/6 background were obtained from Dr. Feron (UC Louvain, Brussels, Belgium). C57BL/6 mice were purchased from Charles River Laboratories. All mice used were between 5 and 13 weeks old, without specific gender selection. In all experiments, littermate controls were used. Housing and all experimental animal procedures were approved by the Institutional Animal Care and Research Advisory Committee of KU Leuven.

### BM Transplantation

6-week-old C56BL/6 recipient mice were lethally irradiated with 9.5 Gy. Subsequently, 10 × 10^6^ BM cells from the appropriate genotype were injected intravenously via tail vein. Tumor experiments were initiated 6–8 weeks after BM reconstitution. Blood cell count was determined using a hemocytometer on peripheral blood collected by retro-orbital bleeding.

### Tumor Models

1 × 10^6^ LLC cells were injected subcutaneously or 5 × 10^5^ E0771 cells were injected orthotopically in the mammary fat pad. Tumor volumes were measured three times a week with a caliper using the formula V = π × d^2^ × D/6, where d is the minor tumor axis and D is the major tumor axis. At the end stage, tumor weight was measured and lung metastasis nodules were contrasted after intratracheal injection of 15% India ink solution or by H&E staining on lung paraffin sections. Superficial metastatic nodules were assessed under a stereomicroscope. Macrophage depletion was achieved by intraperitoneal (i.p.) injection of a loading dose of 250 μL clodronate and control PBS liposomes (ClodronateLiposomes, the Netherlands). 12 hr later, LLC tumor cells were injected subcutaneously followed by another dose of 250 μL liposomes 6 hr after injection. During tumor progression, repeated injections of 250 μL were performed every second day to prevent repopulation of macrophages. MMTV-PyMT spontaneous breast tumors were measured 20 weeks after birth (8 weeks after BM transplantation), twice a week with a caliper, and mice were killed at week 25.

### LLC Lung Colonization Experiments

5 × 10^5^ LLC cells in 200 μL PBS were injected directly in the bloodstream. VEGFR1 inhibition was achieved by i.p. injection of 20 mg/kg mouse anti-VEGFR1 antibodies (clone MF1, Thrombogenics) or isotype IgG control (Sigma-Aldrich) every second day. MMP9 inhibition was achieved by daily gavage injection of 15 mg/kg MMP9 inhibitor II (444293, Millipore), previously diluted in 50% methylcellulose. DC101 treatment was achieved by i.p. injection of 40 mg/kg rat anti-mouse VEGFR2 antibodies (BioXCell) twice a week. Blockage of VEGF-A was achieved by i.p. injection of 40 mg/kg chimeric anti-mouse VEGF-A (clone B20, ThromboGenics) or isotype IgG control (Sigma-Aldrich). Anti-VEGF injections were performed at day 16 and 18 upon LLC tail vein injection, and mice were killed at day 20.

### FACS Analysis and Flow Sorting of Tumor- and Metastasis-Associated Macrophages

Tumor-bearing mice were sacrificed by cervical dislocation, and tumors and macroscopic lung metastasis were harvested. Tumors and metastases were minced in RPMI medium containing 0.1% collagenase type I and 0.2% dispase type I and incubated in the same solution for 30 min at 37°C. The myeloid cell population in the tumor single-cell suspension was stained for CD45, CD11b, and the pan-macrophage marker F4/80. Cells were subsequently washed and resuspended in FACS buffer before FACS analysis or flow sorting by a FACS Verse or FACS Aria III (BD Biosciences), respectively.

### Statistical Analysis

Data entry and all analyses were performed in a blinded fashion. All statistical analyses were performed using GraphPad Prism software. Statistical significance was calculated by two-tailed unpaired t test on two experimental conditions or two-way ANOVA when repeated measures were compared, with p < 0.05 considered statistically significant. All graphs show mean ± SEM values.

## Author Contributions

W.C., G.D.C., and M.W. designed experiments, performed all experiments, acquired data, performed data analysis, interpreted all data, and wrote the manuscript. A.I.O. performed FACS experiments and data analysis. M.E. performed histological stainings and analysis. B.M.C. conducted scientific direction. M.M. designed experiments, conducted scientific direction, interpreted data, and wrote the manuscript.

## Supplemental Information

Document S1. Supplemental Experimental Procedures, Figures S1–S6, and Table S1

Document S2. Article plus Supplemental Information
