# Supplementary material for: Loss of Caveolin-1 in Metastasis-Associated Macrophages Drives Lung Metastatic Growth through Increased Angiogenesis
Source: Cell Rep. 2017 Dec 5;21(10):2842–54. doi: 10.1016/j.celrep.2017.11.034 (PMC5732321; doi:10.1016/j.celrep.2017.11.034)
Supplement: Document S1. Supplemental Experimental Procedures, Figures S1–S6, and Table S1 [file mmc1.pdf]

Cell Reports, Volume *21*

Supplemental Information

Loss of Caveolin-1 in Metastasis-Associated Macrophages Drives Lung Metastatic Growth through Increased Angiogenesis

Ward Celus, Giusy Di Conza, Ana Isabel Oliveira, Manuel Ehling, Bruno M. Costa, Mathias Wenes, and Massimiliano Mazzone

SUPPLEMENTAL INFORMATION

	TUMOR-FREE MICE		TUMOR-BEARING MICE	
	WT→WT	Cav1 KO→WT	WT→WT	Cav1 KO→WT
WBC (K/μL)	12,40 ± 2,93	13,21 ± 3,57	67,25 ± 24,12	72,23 ± 24,01
NEU (%)	5,37 ± 0,95	5,92 ± 0,72	64,69 ± 12,22	69,70 ± 8,77
LYM (%)	87,43 ± 1,67	86,79 ± 2,53	15,50 ± 8,15	15,70 ± 6,61
MON (%)	3,09 ± 0,88	2,94 ± 0,71	3,02 ± 1,87	2,94 ± 1,63
EOS (%)	0,14 ± 0,05	0,21 ± 0,25	0,05 ± 0,04	0,06 ± 0,04
BAS (%)	3,71 ± 0,78	3,93 ± 0,12	3,12 ± 1,01	3,05 ± 0,04
RBC (M/μL)	8,90 ± 0,38	8,85 ± 0,49	4,23 ± 0,76	4,70 ± 0,98
PLT (K/μL)	175,50 ± 75,39	282,34 ± 116,71	180,26 ± 91,54	283,24 ± 78,84

Table S1. Hematological parameters of tumor-free and tumor-bearing WT→WT and Cav1 KO→WT chimeric mice. Related to Figure 1.

The values represent hematological parameters (mean ± SEM) in WT→WT and Cav1 KO→WT chimeric mice, measured before (tumor-free) and 14 days after the implantation of subcutaneous LLC tumors (tumor-bearing). Data are pooled from 2 independent experiments. Total mice, n = 10 per genotype.
Abbreviations: white blood cell (WBC), neutrophil (NEU), lymphocyte (LYM), monocyte (MON), eosinophil (EOS), basophil (BAS), red blood cell (RBC), platelet (PLT).

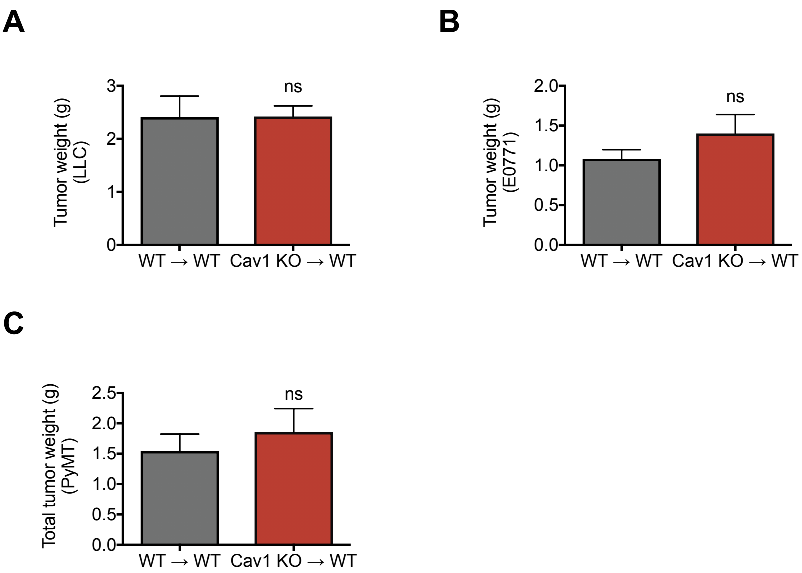

Figure S1. Cav1 deletion in the bone marrow does not affect primary tumor weight in multiple tumor models. Related to Figure 1.

(A) Tumor weight of WT→WT and Cav1 KO→WT chimeric mice in a subcutaneous LLC lung cancer model. Total mice, n = 30 per genotype.
(B) Tumor weight of WT→WT and Cav1 KO→WT chimeric mice in an orthotopic E0771 breast cancer model. Total mice, n = 8 per genotype.
(C) Total tumor weight of lethally irradiated PyMT mice reconstituted with WT or Cav1 KO bone marrow cells. Total mice, n = 7 per genotype.
All graphs show mean ± standard error of the mean (SEM). ns, not significant.

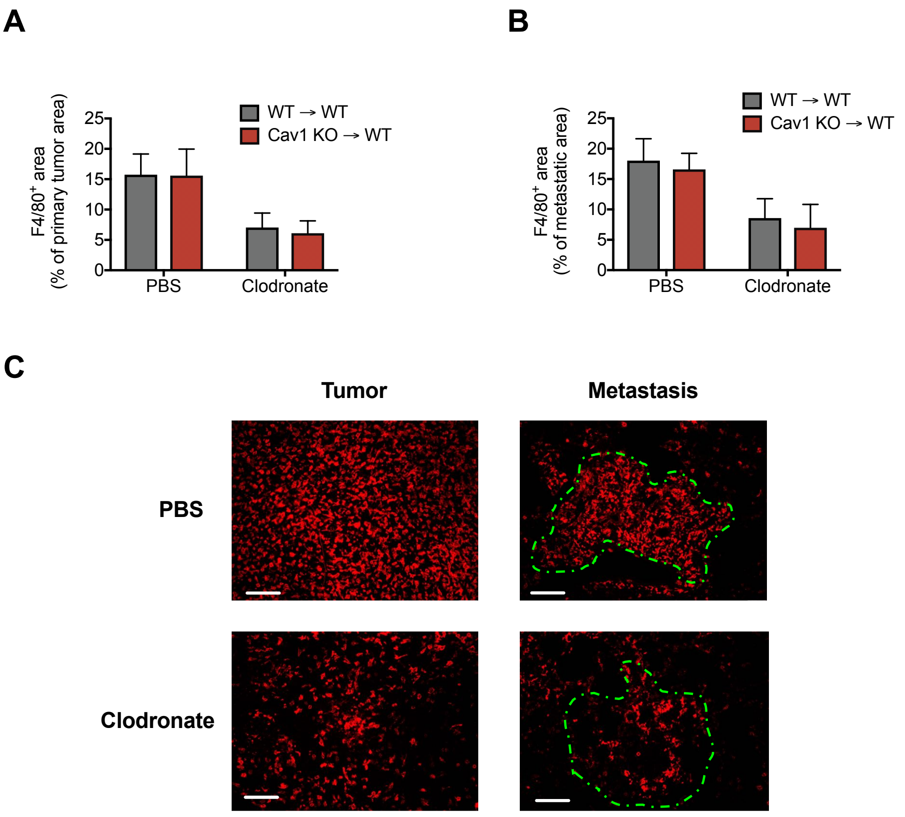

Figure S2. Macrophage infiltration is unaffected in both primary tumor and metastasis upon Cav1 deletion in the bone marrow. Related to Figure 2.

(A-C) Percentage of F4/80$^+$ macrophages upon treatment with macrophage-depleting clodronate liposomes or PBS in LLC primary tumors (A) and LLC metastatic lung lesions (B). Representative pictures of clodronate efficacy in both primary tumor and metastasis (C). Scale bar: 100 µm. Total mice, n = 10 per genotype.
All graphs show mean ± SEM.

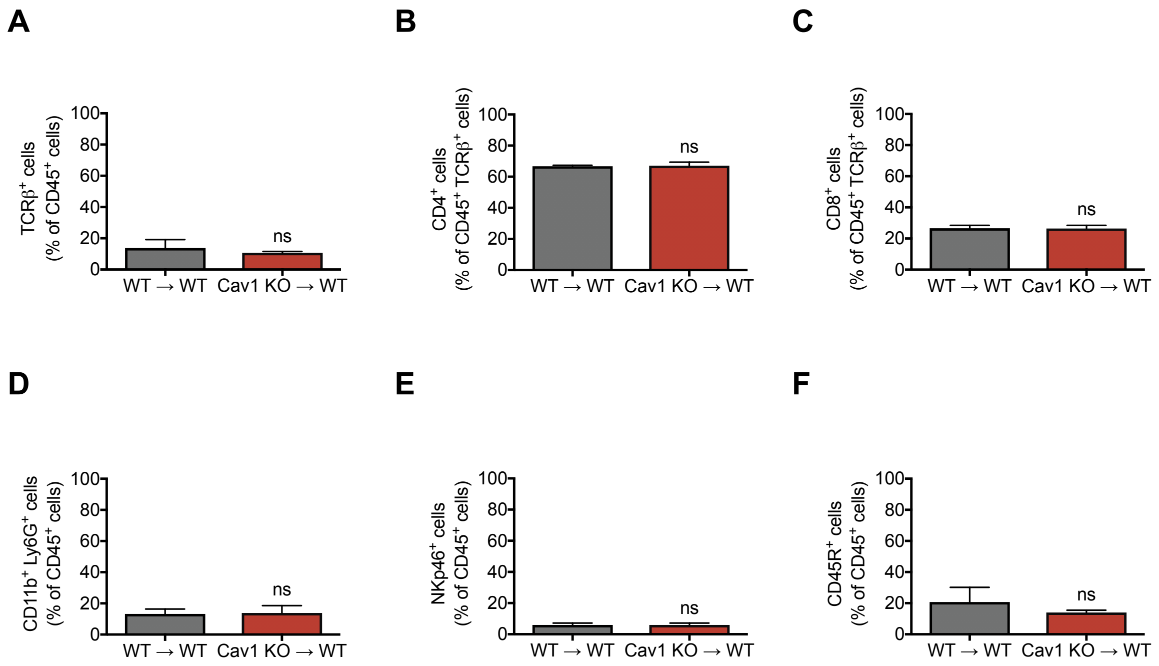

Figure S3. Cav1 deletion in the bone marrow does not affect immune cell infiltration in the metastasis. Related to Figure 4.

(A-F) FACS quantification of total TCRβ^{+} cells (A), CD4^{+} T cells (B), CD8^{+} T cells (C), CD11b^{+}Ly6G^{+} neutrophils (D), NKp46^{+} NK cells (E) and CD45R^{+} B cells (F) in WT and Cav1 KO lung metastatic lesions. Total mice, n = 4 per genotype.

All graphs show mean ± SEM. ns, not significant.

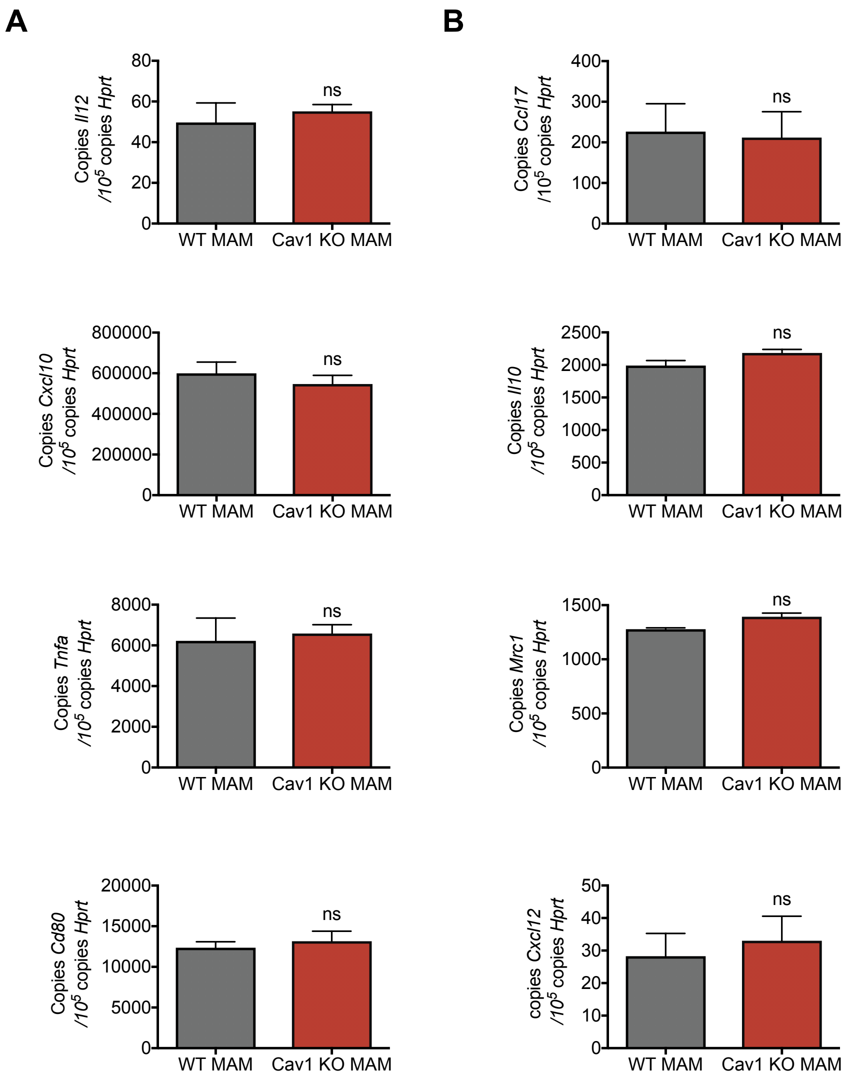

Figure S4. M1-like and M2-like markers are unaffected upon Cav1 deletion in sorted MAMs. Related to Figure 4.

(A and B) qRT-PCR quantification of M1-like markers IL-12, CXCL10, TNFα and CD80 (C) and M2-like markers CCL17, IL-10, Mrc1 and CXCL12 in WT and Cav1 KO sorted MAMs from lung metastatic lesions. Total mice, n = 4 per genotype.

All graphs show mean ± SEM. ns, not significant.

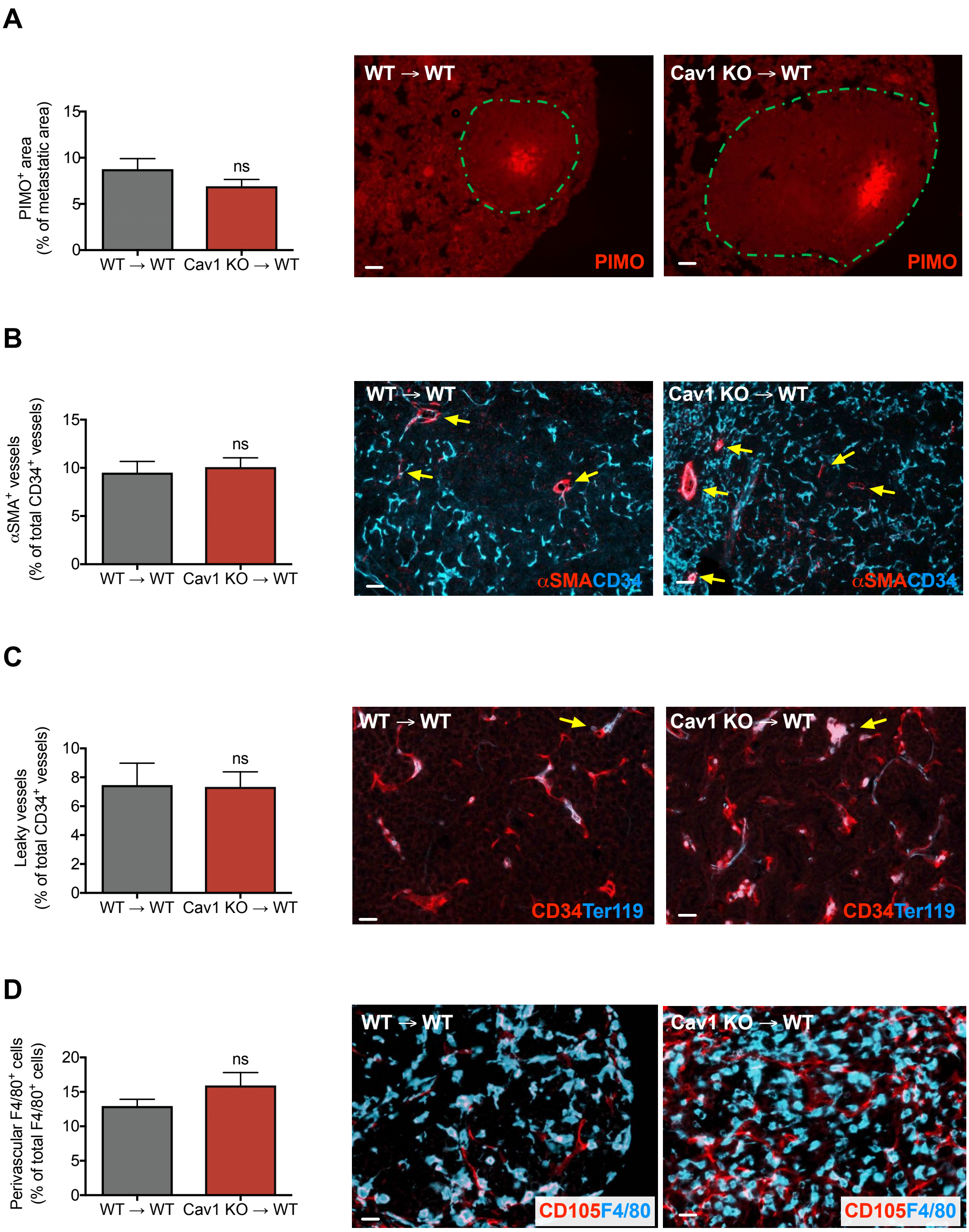

Figure S5. The functionality and vascular integrity of blood vessels in the pulmonary metastases are unchanged upon Cav1 deletion in BMDCs. Related to Figure 4.

(A) Quantification and representative images of PIMO$^+$ (pimonidazole) hypoxic areas in WT and Cav1 KO lung metastatic lesions. Scale bar: 100 µm. Total mice, n = 7 per genotype.

(B) Quantification and representative images of αSMA$^+$ pericyte-covered vessels (arrows) over the total number of CD34+ vessels in WT and Cav1 KO lung metastatic lesions. Scale bar: 50 µm. Total mice, n = 7 per genotype.

(C) Quantification and representative images of leaky vessels in WT and Cav1 KO lung metastatic lesions, measured as the percentage of CD105$^+$ vessels surrounded by Ter119$^+$ red blood cells (arrows) over the total number of vessels. Scale bar: 50 µm. Total mice, n = 7 per genotype.

(D) Quantification and representative pictures of perivascular macrophages measured as the percentage of perivascular F4/80$^+$ macrophages over the total number of F4/80$^+$ macrophages in the metastatic lesion. Scale bar: 50 µm. Total mice, n = 4 per genotype.

All graphs show mean ± SEM. ns, not significant.

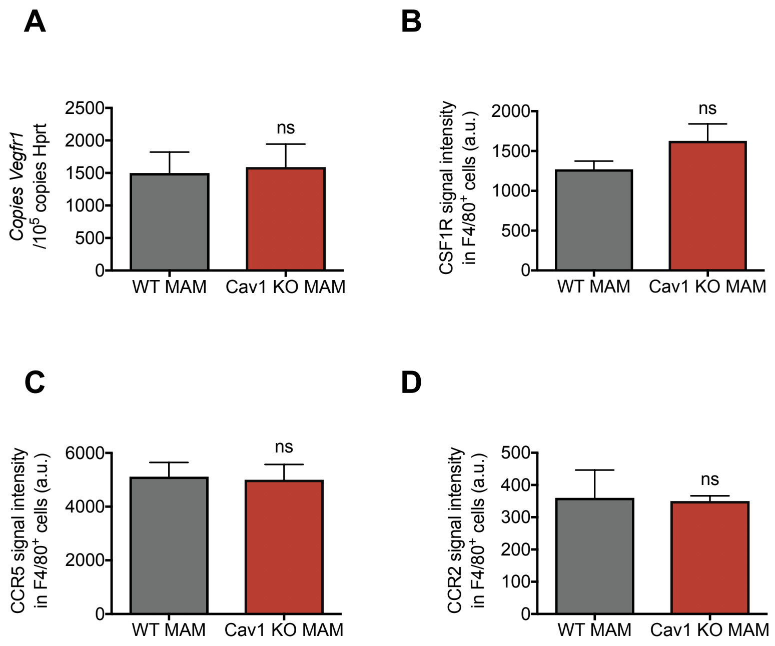

Figure S6. Cav1 deletion in MAMs leads to a specific membrane deficiency in VEGFR1. Related to Figure 5.

(A) *Vegfr1* expression in sorted $CD45^{+}CD11b^{+}F4/80^{+}$ MAMs. Total mice, n = 6 per genotype.
(B-D) FACS analysis of the mean CCR2, CSF-1R and CCR5 signal intensity on $CD45^{+}CD11b^{+}F4/80^{+}$ macrophages in lung metastatic lesions. Total mice, n = 6 per genotype.
All graphs show mean ± SEM. ns, not significant.

SUPPLEMENTAL EXPERIMENTAL PROCEDURES

Animals

Cav1 KO mice on a C57BL/6 background were obtained from Dr. Feron (UCLouvain, Brussels, Belgium). C57BL/6 mice were purchased from Charles River. All mice used were between 5 and 13 weeks old, without specific gender selection. In all experiments, littermate controls were used. Housing and all experimental animal procedures were approved by the Institutional Animal Care and Research Advisory Committee of the KU Leuven.

Bone marrow transplantation

6-week-old C56BL/6 recipient mice were lethally irradiated with 9.5 Gy. Subsequently, 10 x 10^6 bone marrow cells from the appropriate genotype were injected intravenously via tail vein. Tumor experiments were initiated 6 to 8 weeks after bone marrow reconstitution. Red and white blood cell count was determined using a hemocytometer on peripheral blood, collected in heparin with capillary pipettes by retro-orbital bleeding.

Cell lines

Murine Lewis lung carcinoma cells (LLC) and the E0771 medullary breast adenocarcinoma cells were obtained from the American Type Culture Collection (ATCC) and CH3Biosystems, respectively. The cells were cultured in DMEM (Gibco) supplemented with 10% Fetal Bovine Serum (FBS, Gibco), 2 mM glutamine, 100 units/ml penicillin and 100 µg/ml streptomycin. All cells were passaged in the laboratory for no longer than 6 months after receipt.

Tumor models

1 x 10^6 LLC adherent growing murine cells were injected subcutaneously at the right side of the mouse in a volume of 200 µl of PBS. Alternatively, 5 x 10^5 E0771 medullary breast adenocarcinoma cells were injected orthotopically in the mammary fat pad of the second nipple on the right side in a volume of 50 µl of PBS. Tumor volumes were measured three times a week with a caliper and calculated using the formula: $V = \pi \times d^2 \times D/6$, where d is the minor tumor axis and D is the major tumor axis. At the end stage, tumor weight was measured and lung metastasis nodules were contrasted after intratracheal injection of 15% India ink solution or by H&E staining on lung paraffin sections. Superficial metastatic nodules were assessed under a stereomicroscope. MMTV-PyMT spontaneous breast tumors were measured 20 weeks after birth (8 weeks after bone marrow transplantation), twice a week with a caliper, and mice were killed at week 25.

Macrophage depletion

Macrophage depletion was achieved by intraperitoneal injection of a loading dose of 250 µl clodronate and control PBS liposomes, purchased in suspension from ClodLip (ClodronateLiposomes, The Netherlands) (Van Rooijen and Sanders, 1994). 12h later, LLC tumor cells were injected subcutaneously followed by another dose of 250 µl liposomes 6h after injection. During tumor progression, repeated injections of 250 µl every second day to prevent repopulation of macrophages. The efficiency of macrophage depletion was assessed by immunostainings of tumor and lung sections for F4/80.

LLC lung extravasation

To determine the kinetics of tumor cell extravasation in the lung, we transduced LLC cells with a lentivirus expressing a puromycin resistance gene. After puromycin selection, 5 x 10^5 LLC cells in 200 µl PBS were injected directly in the bloodstream. Lungs were dissected at the indicated time points and snap frozen in liquid nitrogen. Whole lungs were homogenized by a ribolyser (MPBio) and gDNA was isolated by the DNeasy Blood and Tissue Kit (Qiagen). The number of LLC cells present in the lungs was measured by qPCR on 2 µg gDNA by using primers against the lentiviral backbone, normalizing with a standard curve containing 2-fold dilutions of the transduced LLC cells.

LLC lung colonization experiments

5 x 10^5 LLC cells in 200 µl PBS were injected directly in the bloodstream. VEGFR1 inhibition was achieved by i.p. injection of 20 mg/kg mouse anti-VEGFR1 antibodies (clone MF1, Thrombogenics) or isotype IgG control (Sigma-Aldrich) every second day. MMP9 inhibition was achieved by daily gavage injection of 15 mg/kg MMP9-inibitor II (444293 from Millipore), previously diluted in 50% methylcellulose. DC101 treatment was achieved by i.p. injection of 40 mg/kg rat anti-mouse VEGFR2 antibodies (BioXcell) twice a week. Blockage of VEGF-A was achieved by i.p. injection of 40 mg/kg chimeric anti-mouse VEGF-A (clone B20, ThromboGenics) or isotype IgG control (Sigma-Aldrich). Anti-VEGF injections were performed at day 16 and 18 upon LLC tail vein injection. For all treatments, mice were dissected after 20 days and lung metastatic nodules were contrasted after intratracheal injection of 15% India ink solution.

Portal vein injection of E0771 breast cancer cells

For intraportal tumor cell injection, laparotomy was performed through a midline incision. Exponentially growing E0771 cultures were trypsinized, washed and resuspended at a concentration of 1×10^5 cells in 100 µl of PBS. The cells were injected in the portal vein using a 30-gauge syringe. Hemorrhagic control was performed using absorbable collagen swabs for compression at the injection site. After injection, the abdominal wall was closed by a suture and animals were allowed to recover on a warming pad in a separate cage until completely conscious. Mice were killed at day 10 and liver metastatic nodules were contrasted by H&E staining on liver paraffin sections (Goddard et al., 2016).

Histology and immunostainings

Serial sections were cut at 7 µm thickness and tissue samples were fixed in 2% PFA overnight at 4°C, dehydrated and embedded in paraffin. Paraffin slides were first rehydrated to further proceed with antigen retrieval in citrate solution (DAKO). If necessary, 0.3% hydrogen peroxide was added to methanol, to block endogenous peroxidases. The sections were blocked with the appropriate serum (DAKO) and incubated overnight with the following antibodies: rat anti-CD31 (BD Pharmingen), rat anti-F4/80 (Serotec), anti-SMA-Cy3 (Sigma Aldrich), rabbit anti-hypoxyprobe (HPI), goat anti-CD105, rat anti-Ter119 (R&D systems) and Hoechst (Thermo Fisher Scientific). Appropriate secondary antibodies were used: Alexa 488, 647 or 568 conjugated secondary antibodies (Molecular Probes), biotin-labeled antibodies (Jackson Immunoresearch) and, when necessary, Tyramide/DAB (Sigma-Aldrich) or TSA Plus Cyanine 3 and Cyanine 5 System amplification (Perkin Elmer, Life Sciences) were performed according to the manufacturer's instructions. Whenever sections were stained in fluorescence, ProLong Gold mounting medium with or without DAPI (Invitrogen) was used. Microscopic analysis was done with an Olympus BX41 microscope and CellSense imaging software. Macrophage and vessel analysis was performed as previously described (Palmieri et al., 2017; Wenes et al., 2016).

Hypoxia assessment

Tumor hypoxia was detected 1h after i.p. injection of 60 mg/kg pimonidazole hydrochloride into tumor-bearing mice. Mice were sacrificed and metastases were harvested. To detect the formation of pimonidazole adducts, metastasis paraffin sections were immunostained with Hypoxyprobe-1-Mab1 (Hypoxyprobe kit, Chemicon) following the manufacturer's instructions.

FACS analysis and flow sorting of tumor- and metastasis-associated macrophages

LLC tumor-bearing mice were sacrificed by cervical dislocation and tumors and macroscopic lung metastasis were harvested. Tumors and metastases were minced in RPMI medium containing 0.1% collagenase type I and 0,2% dispase type I and incubated in the same solution for 30 minutes at 37°C. The digested tissue was filtered using a 70 µm pore sized mesh and cells were centrifuged 5 min at 1000 rpm. Red blood cell lysis was performed by using Hybri-Max^{TM} (Sigma-Aldrich). Cells were resuspended in FACS buffer (PBS containing 2% FBS and 2 mM EDTA) and incubated for 15 minutes with Mouse BD Fc Block purified anti-mouse CD16/CD32 mAb (BD-Pharmingen) and stained with the following antibodies for 30 minutes at 4 °C: Fixable viability dye, anti-CD11b, anti-F4/80, anti-CD11C, anti-CD4, anti-CD8, anti-MHCII, anti-CD206 (Mrc1) (All from Thermo Fisher Scientific), anti-CD45, anti-TCRβ, anti-CD45R (BD Biosciences), anti-FLT1 (R&D Systems), anti-Ly6G, anti-CD335 (NKp46), anti-CCR2, anti-CCR5 and anti-CSF-1R (All from Biolegend). Cells were subsequently washed and resuspended in FACS buffer before FACS analysis or flow sorting by a FACS Verse or FACS Aria III (BD Biosciences), respectively.

Blood and spleen-derived monocytes

For the collection of monocytes, spleen samples were mechanically dissociated, filtered using a 70 µm pore sized mesh and the cells were centrifuged 5 min at 1000 rpm. Blood samples were collected in heparin with capillary pipettes by retro-orbital bleeding. Red blood cell lysis was performed by using Hybri-MaxTM (Sigma-Aldrich). The cells were resuspended in FACS buffer (PBS containing 2% FBS and 2 mM EDTA) and incubated for 15 minutes with Mouse BD Fc Block purified anti-mouse CD16/CD32 mAb (BD-pharmingen) and stained with the following antibodies for 30 minutes at 4 °C: Fixable viability dye, anti-F4/80 (Thermo Fisher Scientific), anti-Ly6C (BD Biosciences) and anti-Ly6G (Biolegend). Cells were subsequently washed and resuspended in cold FACS buffer before flow sorting by a FACS Aria (BD Biosciences). Afterwards, the monocytes were treated for 7 days in culture with 50 ng/ml GM-CSF (Biolegend).

Bone marrow-derived macrophages (BMDMs)

Murine bone marrow-derived macrophages (BMDMs) were derived from bone marrow precursors as described before (Meerpohl et al., 1976). Briefly, bone marrow cells (1,6 x10^6 cells/ml) were cultured in a volume of 6 ml in a 10 cm Petri dish (non-tissue culture treated, bacterial grade) in DMEM supplemented with 20% FBS and 30%

L929 conditioned medium as a source of M-CSF. After 3 days of culture, an additional 3 ml of differentiation medium was added. At day 7, the macrophages were considered differentiated and lysed with RLT buffer (Qiagen) for RNA extraction.

ELISA detection of secreted VEGF-A

The VEGF-A levels in the cell culture medium of sorted MAMs were assessed by ELISA. Following incubation of 2 days, the culture supernatant was collected and VEGF-A levels were measured using a mouse VEGF ELISA kit (Invitrogen, KMG0111) according to the manufacturer's instructions. The absorbance at 450 nm was determined using a microplate reader.

Quantitative RT-PCR

Quantitative RT-PCR was performed as previously described (Fischer et al., 2007), using commercially available (Applied Biosystems and IDT) or homemade primers for the studied genes. The sequence of the primers are available upon request.

MMP9 assay

The gelatinolytic activity of MMP9 was tested using a SDS-page gelatin zymography. Briefly, enzymes were separated based on their molecular weight on 7,5% polyacrylamide-SDS gels containing 1 mg/ml porcine gelatin (Sigma), under non-reducing conditions. For the zymography, a Mini-PROTEAN® III system (Bio-Rad) was used. Protein concentrations of the supernatant from FACS sorted MAMs were determined using a bicinchoninic acid (BCA) Protein Assay Kit (Pierce) according to the manufacturer's instructions. Molecular weight of the gelatinolytic areas was estimated by using Precision Plus Protein Dual Color Standards (Bio-Rad). After migration, gels were washed twice with 2.5% Triton-X100 at room temperature (RT) for 30 min, and incubated with an activation buffer (50 mM Tris-HCl, 10 mM CaCl2, pH 7.5) overnight at 37 °C. Gels were then stained with 0,5% Coomassie Brilliant Blue-R250 for 30 min and destained with a destain buffer (30% methanol, 10% acetic acid) for 60 min at RT. The gelatinolytic activity was visualized as clear bands corresponding to the digested areas. A brighter or a larger band indicated a more concentrated proteinase in the sample. Relative MMP9 activity was analyzed by densitometry using ImageJ.

Statistics

Data entry and all analyses were performed in a blinded fashion. All statistical analyses were performed using GraphPad Prism software on mean values, calculated from the averages of technical replicates. Statistical significance was calculated by two-tailed unpaired t-test on two experimental conditions or two-way ANOVA when repeated measures were compared, with $p < 0.05$ considered statistically significant. Data were tested for normality using the D'Agostino–Pearson omnibus test (for $n > 8$) or the Kolmogorov–Smirnov test (for $n \leq 8$) and variation within each experimental group was assessed. Detection of mathematical outliers was performed using the Grubbs' test in GraphPad. Sample sizes for all experiments were chosen based on previous experiences. Independent experiments were pooled and analyzed together whenever possible. All graphs show mean values ± SEM.

SUPPLEMENTAL REFERENCES

Fischer, C., Jonckx, B., Mazzone, M., Zacchigna, S., Loges, S., Pattarini, L., Chorianopoulos, E., Liesenborghs, L., Koch, M., De Mol, M., *et al.* (2007). Anti-PlGF inhibits growth of VEGF(R)-inhibitor-resistant tumors without affecting healthy vessels. Cell *131*, 463-475.

Goddard, E.T., Fischer, J., and Schedin, P. (2016). A Portal Vein Injection Model to Study Liver Metastasis of Breast Cancer. J Vis Exp.

Meerpohl, H.G., Lohmann-Matthes, M.L., and Fischer, H. (1976). Studies on the activation of mouse bone marrow-derived macrophages by the macrophage cytotoxicity factor (MCF). Eur J Immunol *6*, 213-217.

Palmieri, E.M., Menga, A., Martín-Pérez, R., Quinto, A., Riera-Domingo, C., De Tullio, G., Hooper, D.C., Lamers, W.H., Ghesquière, B., McVicar, D.W., *et al.* (2017). Pharmacologic or Genetic Targeting of Glutamine Synthetase Skews Macrophages toward an M1-like Phenotype and Inhibits Tumor Metastasis. Cell Rep *20*, 1654-1666.

Van Rooijen, N., and Sanders, A. (1994). Liposome mediated depletion of macrophages: mechanism of action, preparation of liposomes and applications. Journal of immunological methods *174*, 83-93.

Wenes, M., Shang, M., Di Matteo, M., Goveia, J., Martín-Pérez, R., Serneels, J., Prenen, H., Ghesquière, B., Carmeliet, P., and Mazzone, M. (2016). Macrophage Metabolism Controls Tumor Blood Vessel Morphogenesis and Metastasis. Cell Metab *24*, 701-715.
