# Supplementary material for: Loss of Caveolin-1 in Metastasis-Associated Macrophages Drives Lung Metastatic Growth through Increased Angiogenesis
Source: Cell Rep. 2017 Dec 5;21(10):2842–54. doi: 10.1016/j.celrep.2017.11.034 (PMC5732321; doi:10.1016/j.celrep.2017.11.034)
Supplement: Document S2. Article plus Supplemental Information [file mmc2.pdf]

Cell Reports

Article

Loss of Caveolin-1 in Metastasis-Associated Macrophages Drives Lung Metastatic Growth through Increased Angiogenesis

Graphical Abstract

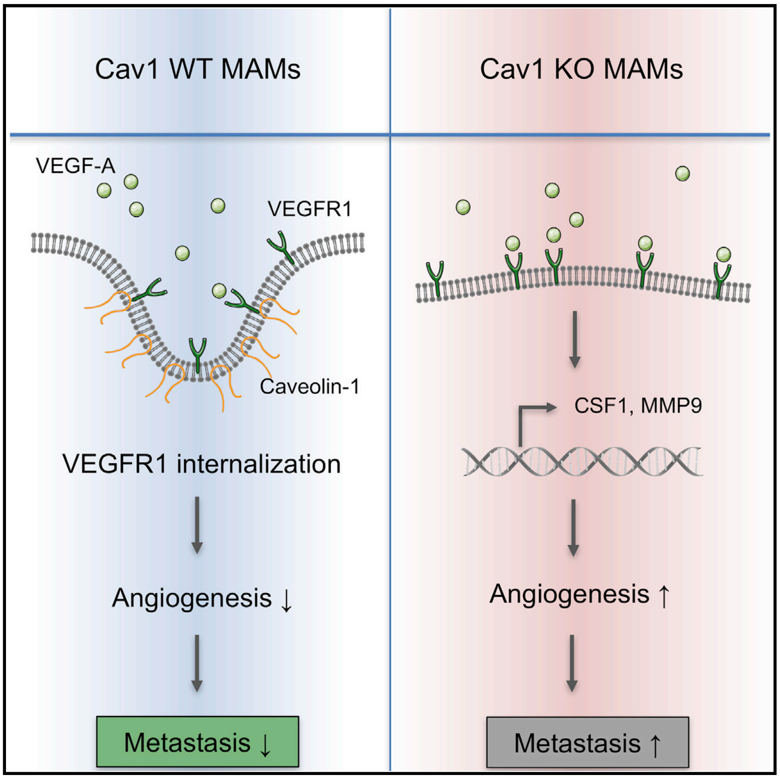

Highlights

- Macrophage Cav1 signaling is critical for restraining lung metastatic growth
- Cav1 deletion in macrophages favors angiogenesis at the lung metastatic site
- Cav1 suppresses VEGF-A/VEGFR1 activity and its downstream effectors, MMP9 and CSF1

Authors

Ward Celus, Giusy Di Conza, Ana Isabel Oliveira, Manuel Ehling, Bruno M. Costa, Mathias Wenes, Massimiliano Mazzone

Correspondence

mathias.wenes@unil.ch (M.W.),
massimiliano.mazzone@kuleuven.vib.be (M.M.)

In Brief

Celus et al. show an intrinsic anti-metastatic surveillance mechanism in the lung microenvironment whereby upregulation of Caveolin-1 in metastasis-associated macrophages specifically controls the excessive exposure of VEGFR1 at the cell surface and thereby limits downstream MMP9 and CSF1 expression, angiogenesis, and metastatic growth.

Celus et al., 2017, Cell Reports *21*, 2842–2854
December 5, 2017 © 2017 The Author(s).
https://doi.org/10.1016/j.celrep.2017.11.034

Check for updates

CellPress

OPEN ACCESS
CellPress

Cell Reports
Article

Loss of Caveolin-1 in Metastasis-Associated Macrophages Drives Lung Metastatic Growth through Increased Angiogenesis

Ward Celus,[1,2,6] Giusy Di Conza,[1,2,6] Ana Isabel Oliveira,[1,2,3,4] Manuel Ehling,[1,2] Bruno M. Costa,[3,4] Mathias Wenes,[1,2,5,*] and Massimiliano Mazzone[1,2,5,7,*]
[1]Lab of Tumor Inflammation and Angiogenesis, Center for Cancer Biology (CCB), VIB, 3000 Leuven, Belgium
[2]Lab of Tumor Inflammation and Angiogenesis, Department of Oncology, KU Leuven, 3000 Leuven, Belgium
[3]Life and Health Sciences Research Institute, School of Medicine, University of Minho, Campus de Gualtar, 4710-057 Braga, Portugal
[4]ICVS/3B's-PT Government Associate Laboratory, Braga/Guimarães, Campus de Gualtar, 4710-057 Braga, Portugal
[5]Senior author
[6]These authors contributed equally
[7]Lead Contact
*Correspondence: mathias.wenes@unil.ch (M.W.), massimiliano.mazzone@kuleuven.vib.be (M.M.)
https://doi.org/10.1016/j.celrep.2017.11.034

SUMMARY

Although it is well established that tumor-associated macrophages take part in each step of cancer progression, less is known about the distinct role of the so-called metastasis-associated macrophages (MAMs) at the metastatic site. Previous studies reported that Caveolin-1 (Cav1) has both tumor-promoting and tumor-suppressive functions. However, the role of Cav1 in bone-marrow-derived cells is unknown. Here, we describe Cav1 as an anti-metastatic regulator in mouse models of lung and breast cancer pulmonary metastasis. Among all the recruited inflammatory cell populations, we show that MAMs uniquely express abundant levels of Cav1. Using clodronate depletion of macrophages, we demonstrate that macrophage Cav1 signaling is critical for metastasis and not for primary tumor growth. In particular, Cav1 inhibition does not affect MAM recruitment to the metastatic site but, in turn, favors angiogenesis. We describe a mechanism by which Cav1 in MAMs specifically restrains vascular endothelial growth factor A/vascular endothelial growth factor receptor 1 (VEGF-A/VEGFR1) signaling and its downstream effectors, matrix metallopeptidase 9 (MMP9) and colony-stimulating factor 1 (CSF1).

INTRODUCTION

During cancer progression, a heterogeneous population of bone-marrow-derived cells (BMDCs) is recruited to the primary tumor site as well as to the pre-metastatic niche or established metastasis (Joyce and Pollard, 2009). Among all BMDCs, macrophages represent the most abundant population and the cells most extensively studied thus far. The plasticity of these cells has long been recognized, and it is known that macrophages carry the potential to not only sustain cancer progression and immunosuppression but also elicit cytotoxicity and antigen presentation and thus immune responses against cancer (Allavena et al., 2008). This opposite response is strictly dependent on the microenvironmental stimuli these cells are subjected to, which can vary from tumor to tumor and from the different niches they occupy (Casazza et al., 2013; Henze and Mazzone, 2016).

Although it is now well established that tumor-associated macrophages (TAMs) take part in each step of cancer growth (Ruffell and Coussens, 2015), less is known about the distinct role of so-called metastasis-associated macrophages (MAMs) at the metastatic site. Several studies have demonstrated different mechanisms through which MAMs support cancer malignancy (Hiratsuka et al., 2002; Qian et al., 2015); however, it is also possible that the immune system induces dormancy in cancer cells that have extravasated to the metastatic site and thus blocks their proliferation and growth (Sosa et al., 2014). Studies in mice using sarcoma, melanoma, and pancreatic cancer models have all pointed to CD4$^+$ T cells and/or CD8$^+$ cytotoxic T cells as the main executors of this metastatic dormancy (Eyles et al., 2010; Koebel et al., 2007; Müller-Hermelink et al., 2008). When looking at the involvement of innate immunity in this process, little is known about the ability of neutrophils and macrophages at the metastatic site to prevent the dissemination or growth of cancer cells (Granot et al., 2011; López-Lago et al., 2013).

Here, we describe how Caveolin-1 (Cav1) in MAMs prevents metastatic growth in the E0771 orthotopic breast cancer model, the subcutaneous Lewis lung carcinoma (LLC) model and the genetically engineered polyoma middle T (MMTV-PyMT) spontaneous breast cancer model. Cav1 is the major component of endocytic caveolae plasma membrane invaginations, and as such, it can control the turnover and activity of several proteins and signaling pathways, such as endothelin B, epidermal growth factor receptor (EGFR), insulin-like growth factor receptor 1 (IGFR-I), and transforming growth factor β (TGF-β) (Couet et al., 1997; He et al., 2015; Ravid et al., 2005; Yamaguchi et al., 2003). In general, Cav1 has been reported to have both

 © 2017 The Author(s).
This is an open access article under the CC BY license (http://creativecommons.org/licenses/by/4.0/).

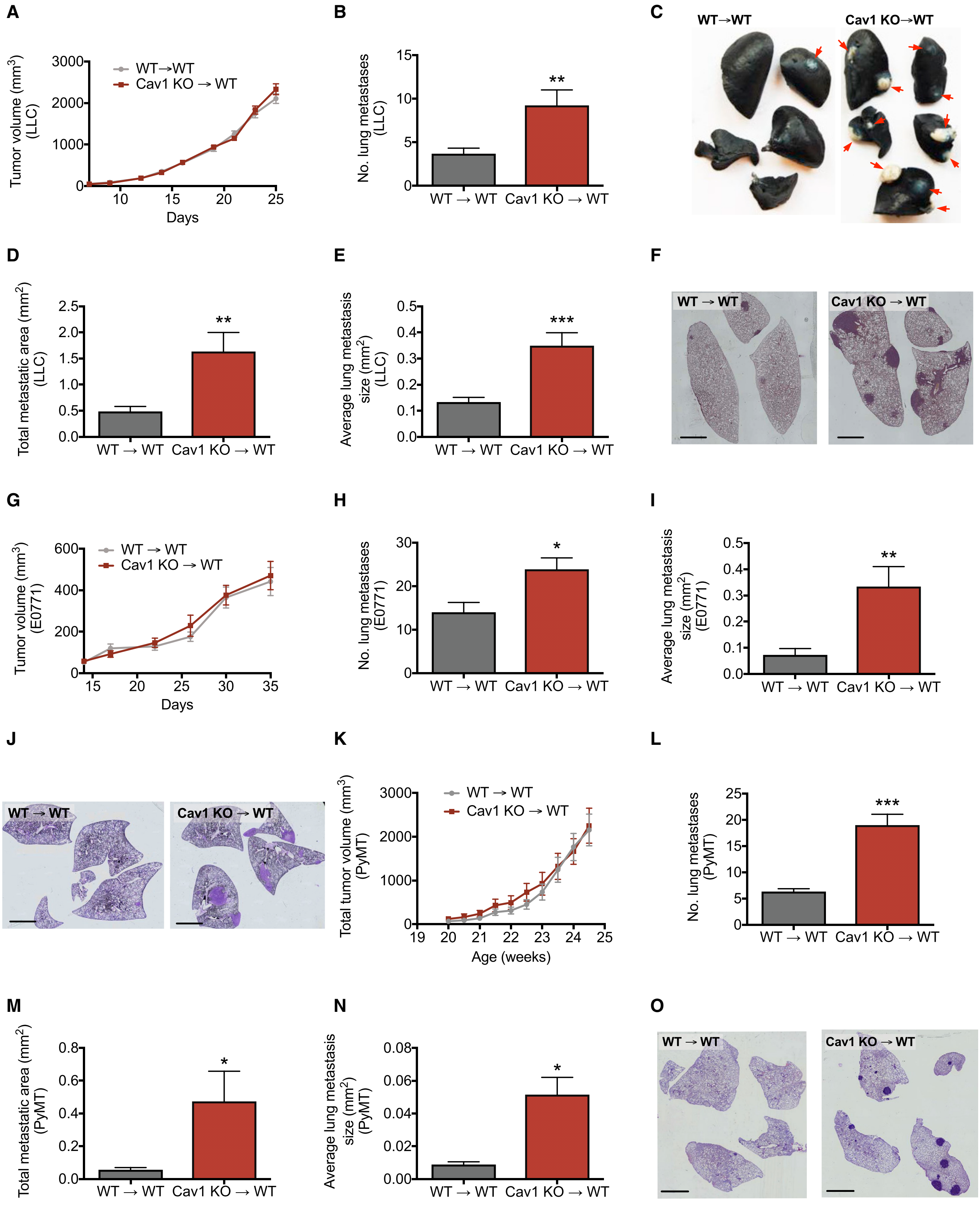

(legend on next page)

tumor-promoting and tumor-suppressive functions, being pro- or anti-survival depending on the cancer cell type (Felicetti et al., 2009; Sunaga et al., 2004; Tanase et al., 2009; Witkiewicz et al., 2010). It is thus clear that the Janus properties of Cav1 are likely due to cell-specific effects, physiological context, and cancer stage. In addition, Cav1 has been characterized in some cell populations of the tumor stroma, where it also shows contrasting effects. For example, in tumor endothelial cells, the expression of Cav1 has been reported to modulate angiogenesis and vascular permeability both positively and negatively (Gratton et al., 2003; Lin et al., 2007; Morais et al., 2012). In cancer-associated fibroblasts, Cav1 underlines matrix stiffness and favors tumor invasion and metastasis (Goetz et al., 2011), whereas others have shown that its loss in fibroblasts correlates with poor prognosis (Capozza et al., 2012; Simpkins et al., 2012). However, the role of Cav1 in BMDCs, particularly macrophages, was never disclosed.

Using chimeric mice, which are Cav1 wild-type (WT) or knockout (KO) in the bone marrow compartment, we describe here a mechanism by which Cav1 in MAMs restrains vascular endothelial growth factor A/vascular endothelial growth factor receptor 1 (VEGF-A/VEGFR1) signaling and its downstream effectors, matrix metallopeptidase 9 (MMP9) and colony-stimulating factor 1 (CSF1). Gene targeting of Cav1 in MAMs unleashes VEGFR1^{+} MAMs and MMP9 activity, which in turn favors the angiogenic switch and metastatic growth. With this in mind, we define here an anti-metastatic function of Cav1 in macrophages.

RESULTS

Deletion of Cav1 in BMDCs Leads to Increased Metastasis in Multiple Tumor Models

In order to investigate the role of Cav1 in BMDCs, we transplanted bone marrow (BM) from WT or *Cav1* KO mice into lethally irradiated WT recipient mice, WT→WT and Cav1 KO→WT, respectively. Upon reconstitution, Cav1 KO→WT chimeras displayed normal blood counts comparable to those of WT→WT mice (Table S1). We then implanted LLC cells subcutaneously. Although primary tumor growth and weight were not different between both groups of chimeric mice (Figures 1A and S1A), Cav1 KO→WT chimeras had 2-fold more lung metastases than WT→WT chimeras (Figures 1B and 1C). Histologically, the total metastatic area was increased upon bone marrow Cav1 deletion, as well as the average size of individual lung metastasis (Figures 1D–1F).

In an alternative tumor model, which was obtained by injecting E0771 breast cancer cells orthotopically in the mammary fat pad, lung metastases were significantly increased upon deletion of Cav1 in the BM, whereas primary tumor growth was similar between groups (Figures 1G, 1H, and S1B). Histological quantifications showed a drastic increase in the average size of individual lung metastasis in Cav1 KO→WT chimeras as compared to WT→WT mice (Figures 1I and 1J). In order to further confirm the anti-metastatic role of Cav1 in BMDCs, we used the polyoma middle T (MMTV-PyMT) spontaneous breast cancer mouse model, which resembles the characteristics of human luminal breast cancer (Lin et al., 2003). Consistent with our previous models, we transplanted the BM from WT or Cav1 KO mice in 12-week-old lethally irradiated PyMT recipient mice (8 weeks before the first tumor onset). We observed that BM *Cav1* deletion in these mice did not affect PyMT primary tumor growth or weight (Figures 1K and S1C). However, the number of metastatic nodules in the lung was much higher in Cav1 KO→WT PyMT chimeras than in WT→WT PyMT controls (Figure 1L). Histological quantifications showed that the total metastatic area, as well as the average size of individual lung metastases, was also increased in PyMT mice upon BM *Cav1* deletion (Figures 1M–1O). Together, these data suggest that Cav1 in BMDCs has an anti-metastatic function in the lungs without affecting primary tumor growth at other sites.

Macrophages Are Responsible for Increased Metastasis in Cav1 KO→WT Chimeras

In tumor-bearing mice, MAMs isolated from lung metastases displayed the highest levels of *Cav1* expression in comparison to TAMs or other immune cells sorted from either the primary tumor or pulmonary metastasis (Figure 2A). Prompted by these data, we evaluated whether Cav1 in macrophages played a role during metastatic growth. Using clodronate liposomes, we achieved an ~55%–60% reduction in macrophage infiltration at the primary tumor and metastatic sites (Figure S2). In this setting, the difference in metastasis between WT→WT and Cav1 KO→WT chimeras was completely abrogated (Figure 2D). At the level of the primary site, clodronate-liposome treatment caused equal tumor growth and weight reduction in both WT→WT and Cav1 KO→WT chimeras (Figures 2B and 2C). However, both TAM and MAM infiltration was not affected by *Cav1* deficiency (Figures 2E, 2F, S2A, and S2B). Overall, these data suggest that Cav1 deletion in macrophages is responsible for increased metastasis.

The Pro-metastatic Function of Cav1 KO Macrophages Is Likely Restricted to the Lungs

Interestingly, when measuring the expression of Cav1 in different tissues of healthy mice, we found that only CD45^{+}CD11b^{+}F4/80^{+}

Figure 1. Deletion of Cav1 in BMDCs Leads to Increased Metastasis in Multiple Tumor Models

(A–F) LLC tumor growth (A) of lethally irradiated WT mice reconstituted with WT (WT→WT) or Cav1 KO (Cav1 KO→WT) BM cells. Quantification of the number of lung metastatic nodules (B) and representative images (C). Metastatic nodules are indicated by arrows. Histological analysis of lung metastasis showing the total metastatic area per mouse (D), average size of each metastasis (E), and representative mosaic images of H&E stained lung sections (F). Scale bar, 2 mm. n = 30 mice per genotype.

(G–J) Tumor growth (G) and number of lung metastatic nodules (H) in an orthotopic E0771 breast cancer model. Histological analysis of lung metastasis showing the average size of each metastasis (I) and representative mosaic images of H&E stained lung sections (J). Scale bar, 2 mm. n = 8 mice per genotype.

(K–O) Total tumor growth (K) and number of lung metastatic nodules (L) of lethally irradiated PyMT mice reconstituted with WT or Cav1 KO BM cells. Histological analysis of lung metastasis showing the total metastatic area per mouse (M), the average size of each metastasis (N), and representative mosaic images of H&E stained lung sections (O). Scale bar, 2 mm. n = 7 mice per genotype.

*$p < 0.05$, **$p < 0.01$, and ***$p < 0.001$ versus WT→WT. All graphs show mean ± SEM.

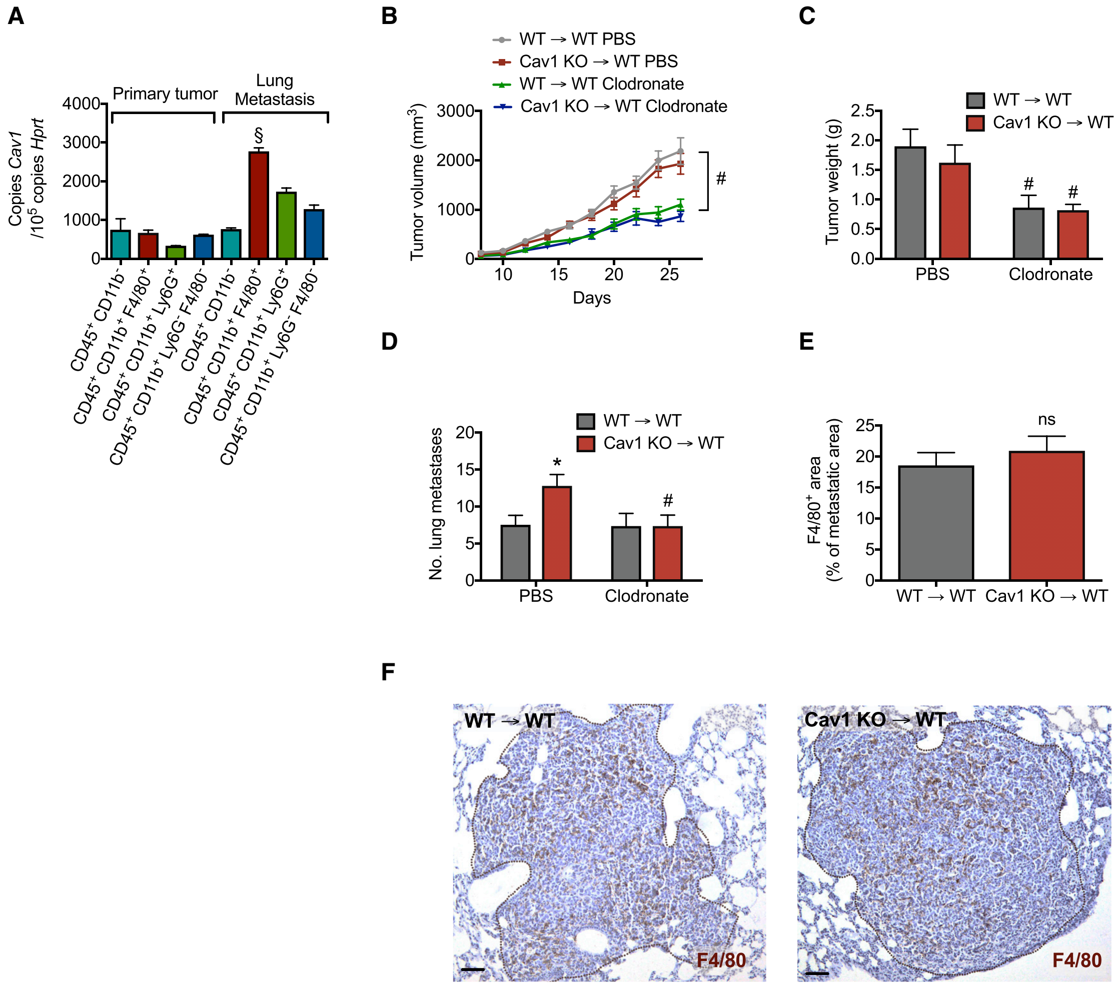

Figure 2. Macrophages Are Responsible for Increased Metastasis in Cav1 KO→WT Chimeras

(A) *Cav1* expression in CD45$^+$CD11b$^-$ non-myeloid cells, CD45$^+$CD11b$^+$F4/80$^+$ macrophages (TAMs), CD45$^+$CD11b$^+$Ly6G$^+$ neutrophils, and other CD45$^+$CD11b$^+$Ly6G$^-$F4/80$^-$ myeloid cells derived from the primary tumor and CD45$^+$CD11b$^-$ non-myeloid cells, CD45$^+$CD11b$^+$F4/80$^+$ macrophages (MAMs), CD45$^+$CD11b$^+$Ly6G$^+$ neutrophils, and other CD45$^+$CD11b$^+$Ly6G$^-$F4/80$^-$ myeloid cells sorted from metastatic nodules of LLC-tumor-bearing mice. n = 4 mice.

(B–D) Tumor growth (B), weight (C), and number of lung metastatic nodules (D) in LLC-tumor-bearing mice treated with macrophage-depleting clodronate liposomes or PBS. n = 11 mice per genotype.

(E and F) F4/80$^+$ macrophage accumulation in LLC metastasis (E) and representative images (F). Scale bar, 100 μm. n = 8 mice per genotype.

§p < 0.05 versus other cell populations, *p < 0.05 versus WT→WT, and $^\#$p < 0.05 versus PBS. ns, not significant. All graphs show mean ± SEM.

interstitial macrophages in the lungs, but not hepatic or bone-marrow-resident macrophages, displayed abundant *Cav1* expression levels (Figure 3A). Fu et al. (2012) previously showed that monocytes treated for 7 days with CSF2 (also known as granulocyte-macrophage colony-stimulating factor [GM-CSF]) displayed increased expression levels of *Cav1*. Consistent with these findings, we observed that 7 days of CSF2 treatment could induce high *Cav1* expression levels in both blood- and spleen-derived Ly-6C$^+$F4/80$^{low/-}$Ly6G$^-$ monocytes (Figure 3B). Downregulation of *Ly6c1* and upregulation of *Erm1* expression upon treatment with CSF2 supports the differentiation of monocytes into macrophages (Figure 3C). These data suggest that upon monocyte arrival to the lungs and during their transition into macrophages, *Cav1* expression can be induced by CSF2, a factor known to play a key role in the homeostatic control of the pulmonary macrophage phenotype (Kopf et al., 2015; Lavin et al., 2015; Mantovani and Allavena, 2015). Based on these observations, we then tested whether the organ context plays a role in the observed phenotype. For this, we examined breast cancer metastasis to the liver by performing portal vein injection of E0771 breast cancer cells (Goddard et al., 2016). After 10 days, we detected no difference in the number of metastatic nodules in the liver between Cav1 KO→WT chimeras and WT→WT mice (Figures 3D–3F). Altogether, we conclude that the effects of *Cav1* deletion in MAMs are likely restricted to the lung parenchyma.

Cav1 Deletion in MAMs Leads to Increased Metastatic Growth and Angiogenesis

Because we observed that MAMs show much higher *Cav1* expression than TAMs (Figure 2A), we hypothesized that the

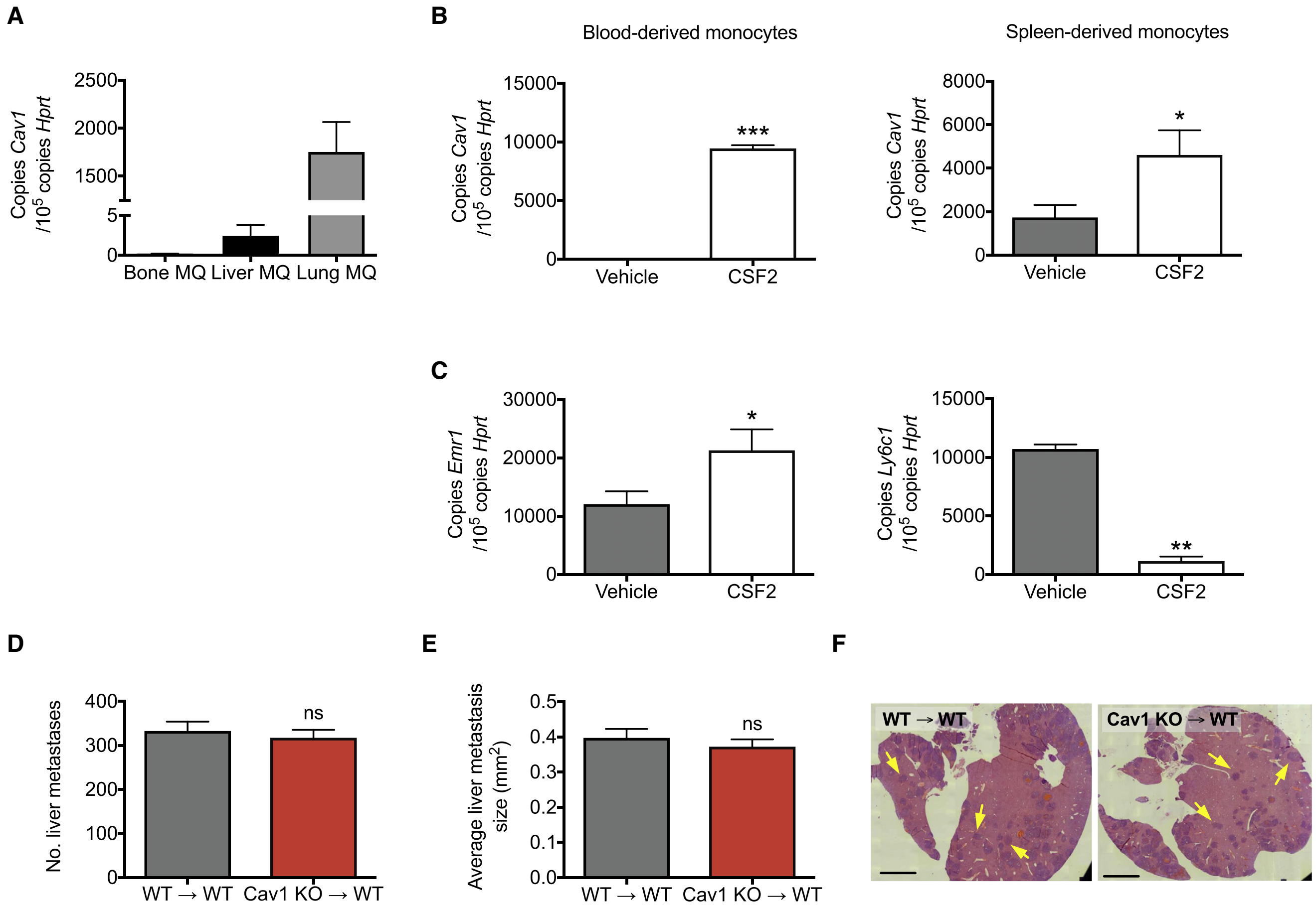

Figure 3. The Pro-metastatic Function of Cav1 KO Macrophages Is Likely Restricted to the Lungs

(A) *Cav1* expression in $CD45^+CD11b^+F4/80^+$ macrophages (MQ) sorted from bone, liver, and lung tissue of healthy mice. n = 10 mice.

(B and C) *Cav1* expression in blood- and spleen-derived monocytes treated for 7 days with GM-CSF (CSF2) or vehicle (B), and expression of *Ly6C1* and *Emr1* at day 7 in spleen-derived monocytes (C). n = 4 mice.

(D–F) Number of liver metastatic nodules upon portal vein injection of E0771 cells (D). Histological analysis of hepatic metastasis showing the average size of each metastasis (E) and representative mosaic images of H&E stained liver sections (F). Metastatic nodules are indicated by arrows. Scale bar, 2 mm. n = 5 mice per genotype. *$p < 0.05$, **$p < 0.01$, and ***$p < 0.001$ versus vehicle. ns, not significant. All graphs show mean ± SEM.

effect of Cav1 on metastasis is independent of the primary tumor. We therefore injected LLC cells directly into the bloodstream. After 20 days, we found a significantly higher number of pulmonary metastatic nodules in Cav1 KO→WT versus WT→WT mice (Figure 4A). While *Cav1* deletion in KO→WT chimeras positively affected metastatic cancer cell growth at a late stage, their extravasation and lodging in the early stage after intravenous cancer cell injection was not affected (Figure 4B). We can therefore argue that Cav1 KO macrophages represent supportive MAMs, which mediate the escape from tumor cell dormancy and increase tumor cell proliferation independently of the primary tumor and initial tumor cell extravasation.

In an attempt to further characterize the phenotype of Cav1 KO macrophages in the metastatic niche, we evaluated features of the MAM infiltrate by fluorescence-activated cell sorting (FACS) and analyzed the expression of the M1-like marker major histocompatibility complex class II (MHC class II) and the M2-like marker CD206 (Mrc1) on the cell membrane of $F4/80^+$ cells (Laoui et al., 2014). The abundance of immune cells in general and specifically of macrophages in the metastasis was comparable in both groups of chimeric mice (Figures 4C and 4D). Additionally, Cav1 KO→WT chimeras displayed similar numbers of MHC class II^{low} and $CD206^{high}$ MAMs as compared to WT→WT controls (Figures 4E and 4F). Additionally, Cav1 KO→WT chimeras had numbers of metastasis-infiltrating $CD4^+$ and $CD8^+$ T cells (TCR-β^+ cells), neutrophils ($CD11b^+Ly6G^+$ cells), natural killer (NK) cells ($NKp46^+$ cells), and B cells ($CD45R^+$ cells) similar to WT→WT mice (Figure S3). Gene expression markers typically used to characterize the classically (M1-like) and alternatively activated (M2-like) macrophage phenotype were also unaltered in MAMs derived from WT→WT and Cav1 KO→WT chimeras (Figure S4), suggesting that deletion of Cav1 does not affect macrophage polarization.

During our attempt to understand the biological reasons underlying the metastatic boost upon hematopoietic deletion of Cav1, we found that vessel density in the metastatic lesion

OPEN ACCESS
CellPress

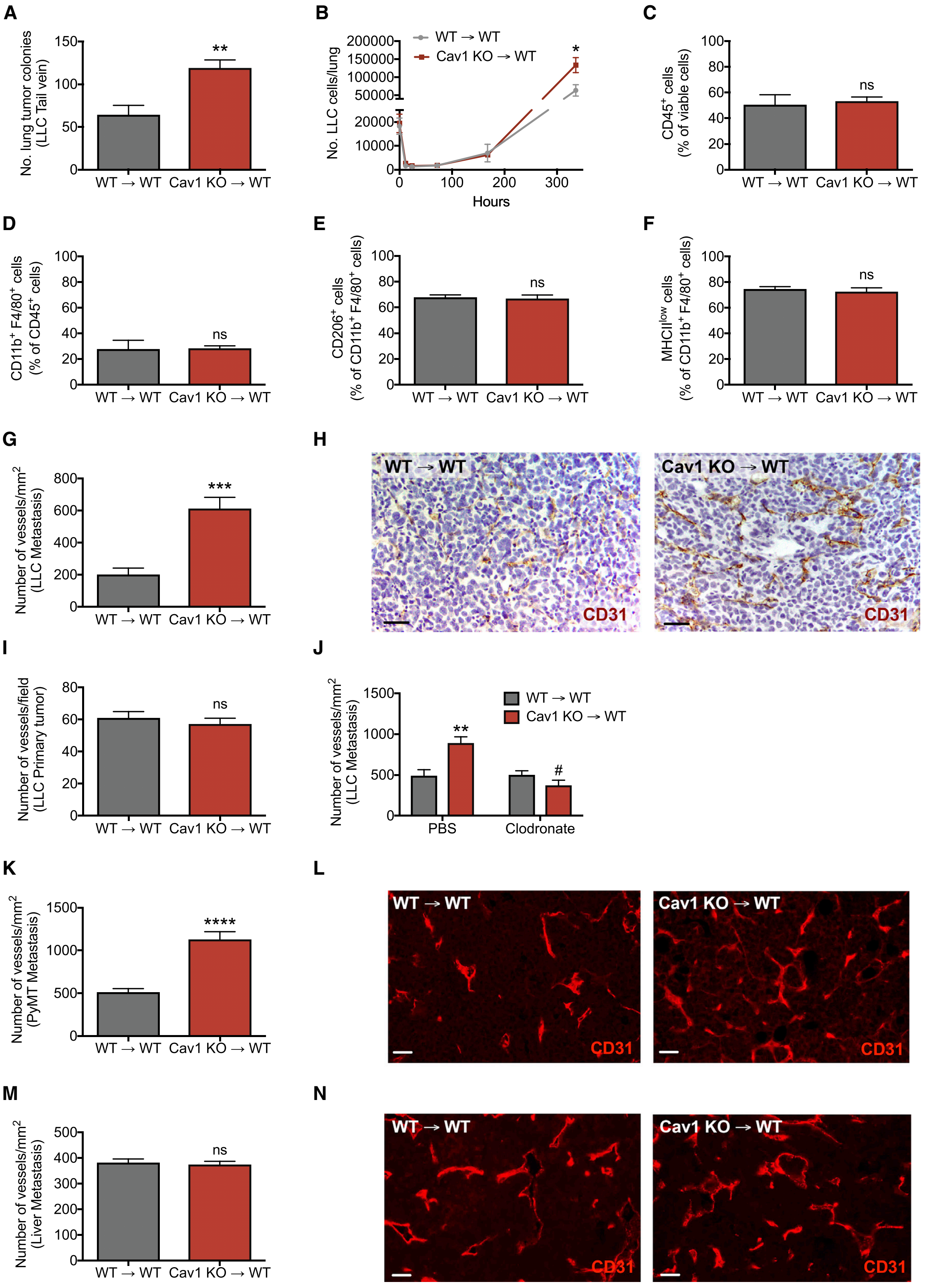

(legend on next page)

was markedly augmented in Cav1 KO→WT versus WT→WT chimeras (Figures 4G and 4H). However, blood vessel density in LLC primary tumors was comparable in both groups of chimeric mice (Figure 4I). The enhanced angiogenesis at the metastatic site in Cav1 KO→WT mice was completely prevented by clodronate-liposome treatment (Figure 4J). Importantly, the observed angiogenic phenotype was consistent in our spontaneous breast cancer model and was organ specific, because PyMT lung metastases in Cav1 KO→WT chimeric mice showed increased blood vessel density, whereas experimental liver metastases did not (Figures 4K–4N).

We also studied the functionality and vascular integrity of the blood vessels in the pulmonary metastases. However, blood vessels in Cav1 KO→WT metastatic lesions did not show any differences in normalization and permeability as compared to WT→WT mice in terms of hypoxic area, pericyte coverage, and number of leaked red blood cells in the perivascular space (Figures S5A–S5C). In addition, the perivascular localization of macrophages in the pulmonary metastatic lesions did not display any significant differences between WT→WT and Cav1 KO→WT mice (Figure S5D). Altogether, these data suggest that MAM-associated Cav1 negatively regulates the sprouting of new blood vessels (but not their function), and this occurs specifically at the lung metastatic site, where Cav1 is abundantly expressed by interstitial macrophages.

Augmented VEGFR1 Activity upon Cav1 Deletion in Macrophages Leads to Increased MMP9-Mediated Metastatic Growth and Angiogenesis

Caveolae are important structural elements involved in modulating signal transduction of several receptors such as VEGFR1, CCR2, or others depending on the cell context (Casalou et al., 2011; Catusse et al., 2009; Dzenko et al., 2001; Fu et al., 2012; Ge and Pachter, 2004). In order to elucidate the mechanism by which *Cav1* deletion leads to increased blood vessel formation, we investigated which cell surface receptors on MAMs are modulated by Cav1. Interestingly, upon *Cav1* deletion, macrophages sorted from metastatic nodules showed increased protein expression of VEGFR1 (also known as Flt1) at the membrane surface (Figure 5A), whereas *Vegfr1* transcripts were the same in both WT and Cav1 KO MAMs (Figure S6A). The upregulation of VEGFR1 in Cav1 KO MAMs was specific, because other cell surface receptors were unaltered (Figures S6B–S6D). Augmented VEGFR1 expression was found only in MAMs, because protein levels of VEGFR1 were similar in WT→WT and Cav1 KO→WT TAMs (Figure 5A). Moreover, we showed that VEGFR1 modulation by Cav1 was responsible for the observed phenotype, because treating mice with the VEGFR1-blocking antibody MF1 abrogated the increased metastatic growth and vessel formation in Cav1 KO→WT chimeras intravenously injected with LLC cells (Figures 5B and 5C). Previous studies have shown that VEGFR1 activity drives *Mmp9* expression in the context of cancer (Bergers et al., 2000; Hiratsuka et al., 2002; Li et al., 2015). Accordingly, we observed a strong increase in *Mmp9* expression in macrophages upon *Cav1* deletion, which was inhibited after MF1 treatment (Figure 5D). In addition, by using an inhibitor of MMP9, we rescued metastatic growth in Cav1 KO→WT mice down to the same levels observed in WT→WT mice (Figure 5E). Accompanying the expression data on *Mmp9*, we observed increased MMP9 activity in sorted MAMs upon deletion of Cav1 (Figure 5F).

Because VEGF-A is the most prominent angiogenic factor secreted by MAMs that stimulates vascular permeability and tumor cell extravasation, we tested the relevance of this ligand in Cav1 KO macrophages (Qian et al., 2011a). We showed that both *Vegfa* expression and secretion of VEGF-A in MAMs were unaffected upon deletion of Cav1 (Figures 5G and 5H). To rule out the possibility that other MMP9-mediated capabilities besides angiogenesis are required for the increased formation of metastasis in Cav1 KO→WT mice, we blocked angiogenesis by injecting DC101 antibodies that, by recognizing VEGFR2, specifically target endothelial cells, but not macrophages (Koch and Claesson-Welsh, 2012). At day 20 upon LLC tail vein injection, DC101 treatment in Cav1 KO→WT chimeras abrogated the increased metastatic growth and angiogenesis to the same levels as control-treated WT→WT mice (Figures 5I–5K), confirming that vessel formation is responsible for the observed phenotype.

Enhanced VEGF-A/VEGFR1 Signaling upon Cav1 Deletion in MAMs Leads to Downstream Activation of the CSF1/MMP9 Axis

VEGF-A and placental growth factor (PlGF) are the two VEGFR1 ligands involved in angiogenesis (Fischer et al., 2008). Of these two ligands, we found that LLC and E0771 cancer cells express high levels of *Vegfa* but barely detectable levels of *Plgf* (Figure 6A). Similar results were obtained when quantifying the amount of *Vegfa* and *Plgf* in LLC-derived lung lesions in WT mice (Figure 6A). Therefore, we decided to block only the VEGF-A ligand and assess how this affects the expression of MMP9. For this approach, WT→WT and Cav1 KO→WT mice were consecutively treated with VEGF-A-blocking antibodies

Figure 4. Cav1 Deletion in MAMs Leads to Increased Metastatic Growth and Angiogenesis
(A) Number of lung metastatic nodules upon tail vein injection of LLC cells. Total mice, n = 6 per genotype.
(B) Tumor cell extravasation measured by qRT-PCR analysis on whole lungs upon tail vein injection of LLC cells. Total mice, n = 4 per condition.
(C–F) FACS quantification of total $CD45^+$ leukocytes (C), $F4/80^+$ MAMs (D), M2-like $CD206^+$ MAMs (E), and MHC class II^{low} MAMs (F) in WT→WT and Cav1 KO→WT lung metastatic lesions. n = 4 mice per genotype.
(G–I) Number of $CD31^+$ blood vessels in LLC metastatic lesions (G) and representative images (H). Scale bar, 50 μm. Number of $CD31^+$ blood vessels in primary LLC tumors (I). n = 10 mice per genotype.
(J) Number of $CD31^+$ blood vessels in LLC metastatic lesions upon treatment with clodronate liposomes or PBS. n = 9 mice per condition.
(K and L) Number of $CD31^+$ blood vessels in lung metastatic lesions of PyMT mice (K) and representative images (L). Scale bar, 50 μm. Total mice, n = 7 per genotype.
(M and N) Number of $CD31^+$ blood vessels in E0771 metastatic lesions in the liver (M) and representative images (N). Scale bar, 50 μm. n = 5 mice per genotype.
$^{**}p < 0.01$, $^{***}p < 0.001$, and $^{****}p < 0.0001$ versus WT→WT. $^{\#}p < 0.05$ versus PBS. ns, not significant. All graphs show mean ± SEM.

OPEN ACCESS
CellPress

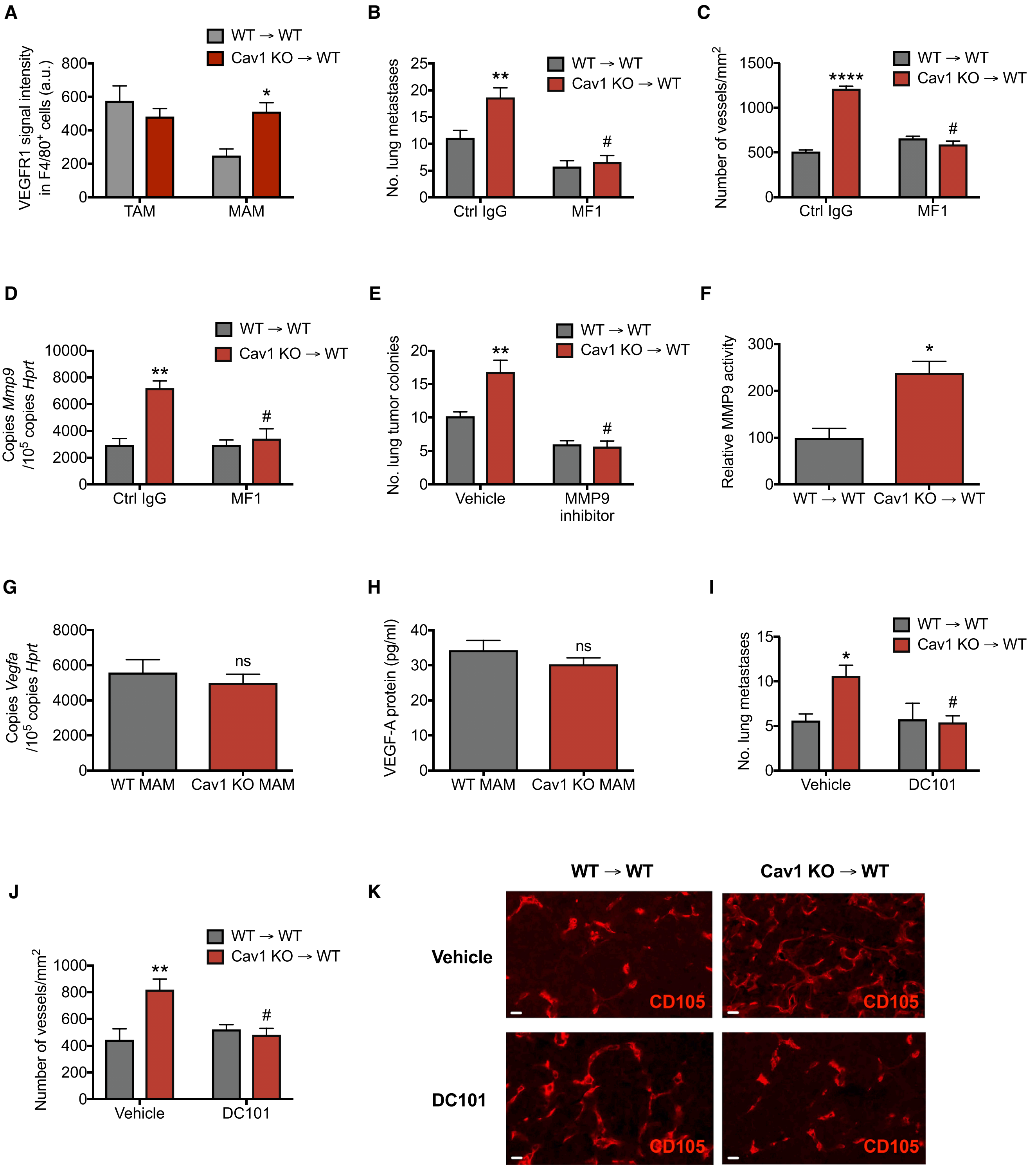

Figure 5. Augmented VEGFR1 Activity upon Cav1 Deletion in Macrophages Leads to Increased MMP9-Mediated Metastatic Growth and Angiogenesis

(A) FACS analysis of the mean VEGFR1 signal intensity on $CD45^+CD11b^+F4/80^+$ macrophages in LLC primary tumors (TAMs) or metastatic lesions (MAMs). n = 4 mice per genotype.

(B–D) Number of lung metastatic nodules upon tail vein injection of LLC cells in mice treated with VEGFR1-blocking antibodies (MF1) or control IgG (Ctrl IgG) (B), and number of $CD105^+$ blood vessels in metastatic lesions (C). *Mmp9* gene expression in FACS-sorted $CD45^+CD11b^+F4/80^+$ MAMs from mice treated with VEGFR1-blocking antibodies (MF1) or control IgG (Ctrl IgG) (D). Total mice, n = 5 per genotype.

(legend continued on next page)

at day 16 and 18 upon LLC tail vein injection. Anti-VEGF-A antibodies were able to rescue metastatic growth in Cav1 KO→ WT mice to the same levels observed in WT→WT mice (Figure 6B). Moreover, *Mmp9* expression in Cav1 KO MAMs treated with anti-VEGF-A decreased to the same levels observed in WT MAMs (Figure 6C), suggesting that VEGFR1-MMP9 signaling upon *Cav1* deletion in MAMs is mainly driven by the VEGF-A ligand.

Qian et al. (2015) have previously shown that CSF1 (also known as macrophage colony-stimulating factor [M-CSF]) is a downstream gene target of VEGFR1 in MAMs. We therefore tested the expression of CSF1 in Cav1 KO MAMs and found that Cav1 KO MAMs display significantly higher *Csf1* expression levels than WT MAMs (Figure 6D). Moreover, MF1 treatment leads to a strong abrogation of *Csf1* expression levels in Cav1 KO MAMs (Figure 6D), suggesting a possible involvement of CSF1 in the Cav1 KO macrophage phenotype. Furthermore, Qian et al. (2015) also showed that macrophage recruitment to the lung was unaffected by MF1 inhibition. We further demonstrated that MAM recruitment was indeed unaffected in either immunoglobulin G (IgG)-treated and MF1-treated Cav1 KO→ WT chimeras (Figure 6E).

In conclusion, these data argue that increased VEGF-A/VEGFR1 activity upon *Cav1* deletion in MAMs drives the downstream expression of MMP9 and CSF1, altogether facilitating blood vessel formation and metastatic growth in the lungs (Figure 6F).

DISCUSSION

Although many studies have thoroughly characterized the role of TAMs and their plastic phenotypes at the primary tumor, far less is known about MAMs in the metastatic niche. Previous studies have shown that circulating monocytes are recruited to the lung metastatic niche and release VEGF that permeabilizes blood vessels, thus enforcing breast cancer cell extravasation (Qian et al., 2011a). Once in the lung parenchyma, metastatic breast cancer cells receive survival signals from MAMs through the interaction of vascular cell adhesion molecule-1 (VCAM-1) and α4-integrins (Chen et al., 2011). Moreover, macrophage VEGFR1 expression, while dispensable for MAM recruitment, is determinant for the regulation of a set of prometastatic genes in MAMs, such as *Csf1* in breast cancer lung metastases (Qian et al., 2015) or *Mmp9* in B16 or LLC lung metastases (Hiratsuka et al., 2002; Kaplan et al., 2005).

In contrast to these metastasis-promoting activities, our data highlight an intrinsic anti-metastatic surveillance mechanism whereby upregulation of Cav1 in MAMs controls excessive expression of VEGFR1, thus limiting expression of MMP9 and CSF1, angiogenesis, and metastatic growth (Figure 6F). These effects are limited to the metastatic niche and were not observed in the primary tumor, possibly because of two reasons. First, MAMs show a much higher expression of Cav1 than TAMs. This might be induced by the lung microenvironment, in which high *Cav1* expression is important to orchestrate appropriate innate immune reactions to pathogens and allergens (Jin et al., 2011). Second, persistent hypoxia (present in the primary tumor) is known to strongly upregulate VEGFR1 expression (Eubank et al., 2011; Okuyama et al., 2006; Roda et al., 2011), thereby possibly saturating any modulatory effects by low *Cav1* expression levels in TAMs. Indeed, hypoxic tumor areas are characterized by the presence of VEGFR1^{high} macrophages that actively secrete MMP9 and thus promote angiogenesis and invasion (Du et al., 2008). Based on our data, we suggest a model in which a VEGFR1-MMP9 signaling axis in lung MAMs is rapidly quenched by high *Cav1* expression, because the treatment of metastasis-bearing mice with the anti-VEGFR1 antibody MF1 did not result in a reduction of *Mmp9* gene expression. On the other hand, KO of Cav1 increased the membrane exposure of VEGFR1 on MAMs, resulting in increased transcription and activity of MMP9, overall inducing excessive blood vessel formation and expanded metastatic size. Nevertheless, a further decrease in lung metastases in WT→WT mice upon MMP9 inhibitor treatment was also observed, which can be explained by the inhibition of MMP9 derived from other cell types, such as endothelial cells (Hiratsuka et al., 2002).

Recent data published by Qian et al. (2015) has demonstrated that MAMs uniquely express VEGFR1 at the cell surface, which is critical for spontaneous lung metastasis, via downstream regulation of a set of inflammatory genes. Among these genes, *Csf1*, a key factor for macrophage survival, was shown to be the main target gene, promoting metastatic growth once cancer cells seed into the lungs. In an effort to link this closely related study with our observed phenotype, we discovered that Cav1 KO MAMs display higher expression levels of *Csf1* than WT MAMs, which were abrogated upon MF1 treatment. These data could indicate that *Cav1* deletion in MAMs could boost the CSF1 inflammatory cascade through excessive exposure of VEGFR1 at the cell surface. Previous studies have shown that blockage of VEGFR1 and CSF1 signaling leads to inhibition of MMP9-mediated actions and consequently suppression of metastatic growth (Kaplan et al., 2005; Priceman et al., 2010). However, further studies are warranted to unravel whether MMP9 and CSF1 act either as two epistatic processes or as one cascade where *Mmp9* expression is downstream CSF1 signaling. When seeking the VEGFR1 ligands responsible for this cascade, we found that VEGF-A, but not placental growth

(E) Number of lung metastatic nodules upon tail vein injection of LLC cells in mice treated with MMP9 inhibitor or vehicle. n = 7 mice per condition.

(F) MMP9 activity in FACS-sorted $CD45^+CD11b^+F4/80^+$ MAMs measured by gelatin zymography. n = 5 mice per genotype.

(G and H) *Vegfa* gene expression in FACS-sorted $CD45^+CD11b^+F4/80^+$ MAMs from WT→WT and Cav1 KO→WT chimeras (G). Protein levels of VEGF-A, measured by ELISA, in the cell supernatants of FACS-sorted $CD45^+CD11b^+F4/80^+$ MAMs from WT→WT and Cav1 KO→WT chimeras (H). n = 4 mice per genotype.

(I–K) Number of lung metastatic nodules upon tail vein injection of LLC cells in mice treated with VEGFR2-blocking antibodies (DC101) or vehicle (I), number of $CD105^+$ blood vessels in lung metastatic lesions (J), and representative images (K). Scale bar, 50 μm. n = 6 mice per condition.

*p < 0.05, **p < 0.01, and ****p < 0.0001 versus WT→WT. #p < 0.05 versus Ctrl IgG or vehicle. ns, not significant. All graphs show mean ± SEM.

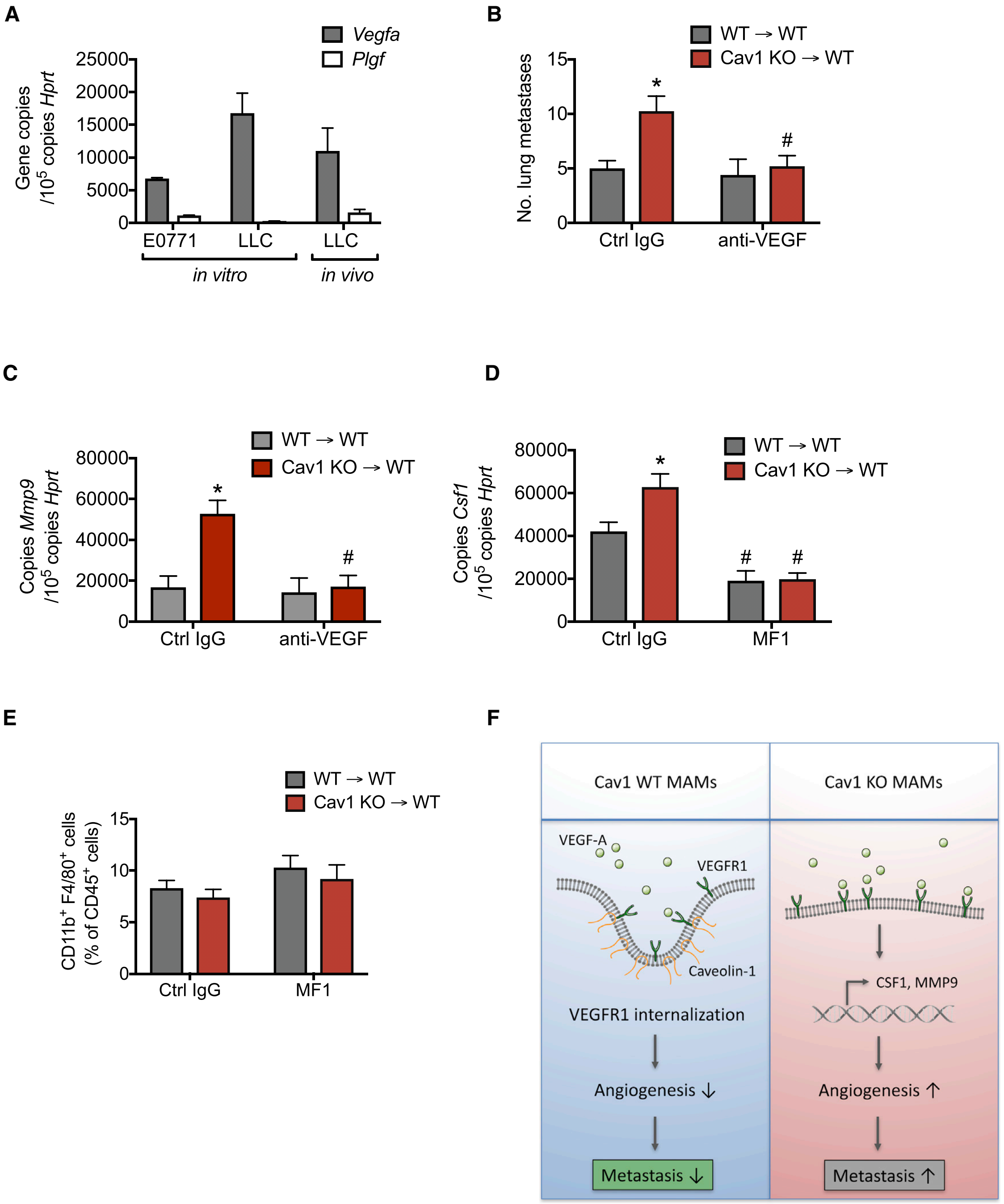

Figure 6. Enhanced VEGF-A/VEGFR1 Signaling upon Cav1 Deletion in MAMs Leads to Downstream Activation of the CSF1/MMP9 Axis

(A) *Vegfa* and *Plgf* expression in E0771 and LLC cancer cells in culture (*in vitro*), and expression of these genes in LLC-derived metastatic lesions (*in vivo*). n = 4 mice.

(B and C) Number of lung metastatic nodules upon tail vein injection of LLC cells in mice treated with anti-VEGF-A antibodies (anti-VEGF) or control IgG (Ctrl IgG) (B) and *Mmp9* gene expression in FACS-sorted $CD45^{+}CD11b^{+}F4/80^{+}$ MAMs (C). n = 4 mice per condition.

(D and E) *Csf1* gene expression in FACS-sorted $CD45^{+}CD11b^{+}F4/80^{+}$ MAMs from mice treated with VEGFR1-blocking antibodies (MF1) or control IgG (Ctrl IgG) (D), and FACS quantification of total $F4/80^{+}$ MAMs (E). n = 5 mice per condition.

(F) Schematic overview of the data showing that deletion of Caveolin-1 (Cav1) in metastasis-associated macrophages (MAMs) drives VEGF-A/VEGFR1 activity and its downstream effectors, MMP9 and CSF1, which in turn favors the angiogenic switch and metastatic growth. Wild-type (WT) MAMs control the excessive exposure of VEGFR1 at the membrane surface via Cav1-mediated internalization, leading to a decrease in the levels of *Mmp9* and *Csf1* transcripts, which results in inhibition of metastatic growth and angiogenesis.

*$p < 0.05$ versus WT→WT. $^{\#}p < 0.05$ versus Ctrl IgG. All graphs show mean ± SEM.

factor (PlGF), was expressed both *in vivo* and *in vitro*, at least in our tumor models. Together with this finding, the use of VEGF-A-blocking antibodies supports the idea that VEGFR1-MMP9 signaling upon *Cav1* deletion in MAMs is mainly driven by VEGF-A.

In the attempt to further characterize the mechanism of Cav1 signaling, we observed that monocytes, treated for 7 days with CSF2 (GM-CSF), display significantly higher *Cav1* expression levels when differentiated into mature macrophages. These data suggest that CSF2 can induce high *Cav1* expression levels in monocyte-derived macrophage subsets, such as interstitial macrophages in the lung. In this context, we can hypothesize that CSF2 might block VEGFR1-CSF1 signaling in a Cav1-dependent manner and thus suppress MMP9-CSF1 pro-metastatic signaling. These data are in agreement with previously proposed competition models between the actions of CSF1 and CSF2 (Fleetwood et al., 2007; Hamilton, 2008). However, further *in vivo* experiments are required to support the validity of this concept.

Another important aspect of this study relates to the organ specificity of the observed phenotype, meaning that the effects of *Cav1* deletion in MAMs are limited to the lung parenchyma as compared to other metastatic sites, such as the liver. We showed that, upon portal vein injection of E0771 breast cancer cells, metastatic growth and vessel density of liver metastatic lesions was unaffected upon BM deletion of Cav1. In addition, the use of subcutaneous LLCs, orthotopic E0771, and transgenic PyMT breast tumors, which all developed larger spontaneous lung metastasis in Cav1 KO→WT mice, allows us to further speculate on the results obtained upon intravenous injection of LLC cells, which also showed enhanced metastatic growth in Cav1 KO→WT mice. Because the latter is a model of lung cancer cells colonizing the pulmonary tissue, previous work has referred to this approach as an orthotopic model of primary lung tumor growth (Goto et al., 2002; Moncho-Amor et al., 2011; Ottewell et al., 2006). Therefore, it is licit to think that Cav1 in macrophages might play a suppressive role during both primary lung carcinogenesis and lung metastasis. However, *in vivo* models of lung carcinogenesis are required to validate this hypothesis.

In conclusion, the role of Cav1 has been extensively studied in cancer, with a focus on cancer cells, endothelial cells, and fibroblasts, leading to the suggestion that the Caveolin scaffolding domain can be used in the treatment of cancer (Gratton et al., 2003; Simpkins et al., 2012; Williams et al., 2004). The therapeutic administration of Cav1 scaffold protein might be a worthwhile pursuit, because the loss of Cav1 in the tumor stroma has been previously associated with more aggressive disease and poor patient outcomes in breast cancer, pancreatic cancer, and melanoma (Jia et al., 2014; Qian et al., 2011b; Wu et al., 2011). We complemented these observations by studying the role of Cav1 in immune cells and thus disclosed an anti-metastatic protective function of Cav1 that is exclusive to MAMs, but not other immune compartments.

EXPERIMENTAL PROCEDURES

More detailed methods can be found in Supplemental Experimental Procedures.

Animals

Cav1 KO mice on a C57BL/6 background were obtained from Dr. Feron (UC Louvain, Brussels, Belgium). C57BL/6 mice were purchased from Charles River Laboratories. All mice used were between 5 and 13 weeks old, without specific gender selection. In all experiments, littermate controls were used. Housing and all experimental animal procedures were approved by the Institutional Animal Care and Research Advisory Committee of KU Leuven.

BM Transplantation

6-week-old C56BL/6 recipient mice were lethally irradiated with 9.5 Gy. Subsequently, 10×10^6 BM cells from the appropriate genotype were injected intravenously via tail vein. Tumor experiments were initiated 6–8 weeks after BM reconstitution. Blood cell count was determined using a hemocytometer on peripheral blood collected by retro-orbital bleeding.

Tumor Models

1×10^6 LLC cells were injected subcutaneously or 5×10^5 E0771 cells were injected orthotopically in the mammary fat pad. Tumor volumes were measured three times a week with a caliper using the formula $V = \pi \times d^2 \times D/6$, where d is the minor tumor axis and D is the major tumor axis. At the end stage, tumor weight was measured and lung metastasis nodules were contrasted after intratracheal injection of 15% India ink solution or by H&E staining on lung paraffin sections. Superficial metastatic nodules were assessed under a stereomicroscope. Macrophage depletion was achieved by intraperitoneal (i.p.) injection of a loading dose of 250 μL clodronate and control PBS liposomes (ClodronateLiposomes, the Netherlands). 12 hr later, LLC tumor cells were injected subcutaneously followed by another dose of 250 μL liposomes 6 hr after injection. During tumor progression, repeated injections of 250 μL were performed every second day to prevent repopulation of macrophages. MMTV-PyMT spontaneous breast tumors were measured 20 weeks after birth (8 weeks after BM transplantation), twice a week with a caliper, and mice were killed at week 25.

LLC Lung Colonization Experiments

5×10^5 LLC cells in 200 μL PBS were injected directly in the bloodstream. VEGFR1 inhibition was achieved by i.p. injection of 20 mg/kg mouse anti-VEGFR1 antibodies (clone MF1, Thrombogenics) or isotype IgG control (Sigma-Aldrich) every second day. MMP9 inhibition was achieved by daily gavage injection of 15 mg/kg MMP9 inhibitor II (444293, Millipore), previously diluted in 50% methylcellulose. DC101 treatment was achieved by i.p. injection of 40 mg/kg rat anti-mouse VEGFR2 antibodies (BioXCell) twice a week. Blockage of VEGF-A was achieved by i.p. injection of 40 mg/kg chimeric anti-mouse VEGF-A (clone B20, ThromboGenics) or isotype IgG control (Sigma-Aldrich). Anti-VEGF injections were performed at day 16 and 18 upon LLC tail vein injection, and mice were killed at day 20.

FACS Analysis and Flow Sorting of Tumor- and Metastasis-Associated Macrophages

Tumor-bearing mice were sacrificed by cervical dislocation, and tumors and macroscopic lung metastasis were harvested. Tumors and metastases were minced in RPMI medium containing 0.1% collagenase type I and 0.2% dispase type I and incubated in the same solution for 30 min at 37°C. The myeloid cell population in the tumor single-cell suspension was stained for CD45, CD11b, and the pan-macrophage marker F4/80. Cells were subsequently washed and resuspended in FACS buffer before FACS analysis or flow sorting by a FACS Verse or FACS Aria III (BD Biosciences), respectively.

Statistical Analysis

Data entry and all analyses were performed in a blinded fashion. All statistical analyses were performed using GraphPad Prism software. Statistical significance was calculated by two-tailed unpaired t test on two experimental conditions or two-way ANOVA when repeated measures were compared, with $p < 0.05$ considered statistically significant. All graphs show mean ± SEM values.

SUPPLEMENTAL INFORMATION

Supplemental Information includes Supplemental Experimental Procedures, six figures, and one table and can be found with this article online at https://doi.org/10.1016/j.celrep.2017.11.034.

AUTHOR CONTRIBUTIONS

W.C., G.D.C., and M.W. designed experiments, performed all experiments, acquired data, performed data analysis, interpreted all data, and wrote the manuscript. A.I.O. performed FACS experiments and data analysis. M.E. performed histological stainings and analysis. B.M.C. conducted scientific direction. M.M. designed experiments, conducted scientific direction, interpreted data, and wrote the manuscript.

ACKNOWLEDGMENTS

The authors thank J. Serneels, R. Kroes, and S. Willox for their technical assistance. We thank ThromboGenics (Leuven, Belgium) for providing the MF1 and anti-VEGF antibodies. Cav1 KO mice were kindly offered by Dr. Feron (UC Louvain, Brussels, Belgium). W.C. was supported by an FWO-Strategic Basic Research (SB) doctoral fellowship (1S26917N), G.D.C. by a Pegasus FWO-Marie Curie fellowship (12114113N), A.I.O. by FCT Portugal (SFRH/BD/52287/2013), and M.E. by the DFG (EH 472/1-1) and Kom op tegen Kanker (Stand up to Cancer), the Flemish cancer society (2016/10538/2453). B.M.C. was funded by FCT Portugal (IF/00601/2012). M.M. received an ERC starting grant (OxyMO, 308459) and long-term structural Methusalem funding by the Flemish government (METH.14.08).

Received: April 18, 2017
Revised: September 22, 2017
Accepted: November 10, 2017
Published: December 5, 2017

REFERENCES

Allavena, P., Sica, A., Garlanda, C., and Mantovani, A. (2008). The Yin-Yang of tumor-associated macrophages in neoplastic progression and immune surveillance. Immunol. Rev. *222*, 155–161.

Bergers, G., Brekken, R., McMahon, G., Vu, T.H., Itoh, T., Tamaki, K., Tanzawa, K., Thorpe, P., Itohara, S., Werb, Z., and Hanahan, D. (2000). Matrix metalloproteinase-9 triggers the angiogenic switch during carcinogenesis. Nat. Cell Biol. *2*, 737–744.

Capozza, F., Trimmer, C., Castello-Cros, R., Katiyar, S., Whitaker-Menezes, D., Follenzi, A., Crosariol, M., Llaverias, G., Sotgia, F., Pestell, R.G., and Lisanti, M.P. (2012). Genetic ablation of Cav1 differentially affects melanoma tumor growth and metastasis in mice: role of Cav1 in Shh heterotypic signaling and transendothelial migration. Cancer Res. *72*, 2262–2274.

Casalou, C., Costa, A., Carvalho, T., Gomes, A.L., Zhu, Z., Wu, Y., and Dias, S. (2011). Cholesterol regulates VEGFR-1 (FLT-1) expression and signaling in acute leukemia cells. Mol. Cancer Res. *9*, 215–224.

Casazza, A., Laoui, D., Wenes, M., Rizzolio, S., Bassani, N., Mambretti, M., Deschoemaeker, S., Van Ginderachter, J.A., Tamagnone, L., and Mazzone, M. (2013). Impeding macrophage entry into hypoxic tumor areas by Sema3A/Nrp1 signaling blockade inhibits angiogenesis and restores antitumor immunity. Cancer Cell *24*, 695–709.

Catusse, J., Clark, D.J., and Gompels, U.A. (2009). CCR5 signalling, but not DARC or D6 regulatory, chemokine receptors are targeted by herpesvirus U83A chemokine which delays receptor internalisation via diversion to a caveolin-linked pathway. J. Inflamm. (Lond.) *6*, 22.

Chen, Q., Zhang, X.H., and Massagué, J. (2011). Macrophage binding to receptor VCAM-1 transmits survival signals in breast cancer cells that invade the lungs. Cancer Cell *20*, 538–549.

Couet, J., Sargiacomo, M., and Lisanti, M.P. (1997). Interaction of a receptor tyrosine kinase, EGF-R, with caveolins. Caveolin binding negatively regulates tyrosine and serine/threonine kinase activities. J. Biol. Chem. *272*, 30429–30438.

Du, R., Lu, K.V., Petritsch, C., Liu, P., Ganss, R., Passegué, E., Song, H., Vandenberg, S., Johnson, R.S., Werb, Z., and Bergers, G. (2008). HIF1alpha induces the recruitment of bone marrow-derived vascular modulatory cells to regulate tumor angiogenesis and invasion. Cancer Cell *13*, 206–220.

Dzenko, K.A., Andjelkovic, A.V., Kuziel, W.A., and Pachter, J.S. (2001). The chemokine receptor CCR2 mediates the binding and internalization of monocyte chemoattractant protein-1 along brain microvessels. J. Neurosci. *21*, 9214–9223.

Eubank, T.D., Roda, J.M., Liu, H., O'Neil, T., and Marsh, C.B. (2011). Opposing roles for HIF-1α and HIF-2α in the regulation of angiogenesis by mononuclear phagocytes. Blood *117*, 323–332.

Eyles, J., Puaux, A.L., Wang, X., Toh, B., Prakash, C., Hong, M., Tan, T.G., Zheng, L., Ong, L.C., Jin, Y., et al. (2010). Tumor cells disseminate early, but immunosurveillance limits metastatic outgrowth, in a mouse model of melanoma. J. Clin. Invest. *120*, 2030–2039.

Felicetti, F., Parolini, I., Bottero, L., Fecchi, K., Errico, M.C., Raggi, C., Biffoni, M., Spadaro, F., Lisanti, M.P., Sargiacomo, M., and Carè, A. (2009). Caveolin-1 tumor-promoting role in human melanoma. Int. J. Cancer *125*, 1514–1522.

Fischer, C., Mazzone, M., Jonckx, B., and Carmeliet, P. (2008). FLT1 and its ligands VEGFB and PlGF: drug targets for anti-angiogenic therapy? Nat. Rev. Cancer *8*, 942–956.

Fleetwood, A.J., Lawrence, T., Hamilton, J.A., and Cook, A.D. (2007). Granulocyte-macrophage colony-stimulating factor (CSF) and macrophage CSF-dependent macrophage phenotypes display differences in cytokine profiles and transcription factor activities: implications for CSF blockade in inflammation. J. Immunol. *178*, 5245–5252.

Fu, Y., Moore, X.L., Lee, M.K., Fernández-Rojo, M.A., Parat, M.O., Parton, R.G., Meikle, P.J., Sviridov, D., and Chin-Dusting, J.P. (2012). Caveolin-1 plays a critical role in the differentiation of monocytes into macrophages. Arterioscler. Thromb. Vasc. Biol. *32*, e117–e125.

Ge, S., and Pachter, J.S. (2004). Caveolin-1 knockdown by small interfering RNA suppresses responses to the chemokine monocyte chemoattractant protein-1 by human astrocytes. J. Biol. Chem. *279*, 6688–6695.

Goddard, E.T., Fischer, J., and Schedin, P. (2016). A portal vein injection model to study liver metastasis of breast cancer. J. Vis. Exp. *118*, 54903.

Goetz, J.G., Minguet, S., Navarro-Lérida, I., Lazcano, J.J., Samaniego, R., Calvo, E., Tello, M., Osteso-Ibáñez, T., Pellinen, T., Echarri, A., et al. (2011). Biomechanical remodeling of the microenvironment by stromal caveolin-1 favors tumor invasion and metastasis. Cell *146*, 148–163.

Goto, H., Yano, S., Zhang, H., Matsumori, Y., Ogawa, H., Blakey, D.C., and Sone, S. (2002). Activity of a new vascular targeting agent, ZD6126, in pulmonary metastases by human lung adenocarcinoma in nude mice. Cancer Res. *62*, 3711–3715.

Granot, Z., Henke, E., Comen, E.A., King, T.A., Norton, L., and Benezra, R. (2011). Tumor entrained neutrophils inhibit seeding in the premetastatic lung. Cancer Cell *20*, 300–314.

Gratton, J.P., Lin, M.I., Yu, J., Weiss, E.D., Jiang, Z.L., Fairchild, T.A., Iwakiri, Y., Groszmann, R., Claffey, K.P., Cheng, Y.C., and Sessa, W.C. (2003). Selective inhibition of tumor microvascular permeability by cavtratin blocks tumor progression in mice. Cancer Cell *4*, 31–39.

Hamilton, J.A. (2008). Colony-stimulating factors in inflammation and autoimmunity. Nat. Rev. Immunol. *8*, 533–544.

He, K., Yan, X., Li, N., Dang, S., Xu, L., Zhao, B., Li, Z., Lv, Z., Fang, X., Zhang, Y., and Chen, Y.G. (2015). Internalization of the TGF-β type I receptor into caveolin-1 and EEA1 double-positive early endosomes. Cell Res. *25*, 738–752.

Henze, A.T., and Mazzone, M. (2016). The impact of hypoxia on tumor-associated macrophages. J. Clin. Invest. *126*, 3672–3679.

Hiratsuka, S., Nakamura, K., Iwai, S., Murakami, M., Itoh, T., Kijima, H., Shipley, J.M., Senior, R.M., and Shibuya, M. (2002). MMP9 induction by vascular

endothelial growth factor receptor-1 is involved in lung-specific metastasis. Cancer Cell *2*, 289–300.

Jia, Y., Wang, N., Wang, J., Tian, H., Ma, W., Wang, K., Tan, B., Zhang, G., Yang, S., Bai, B., and Cheng, Y. (2014). Down-regulation of stromal caveolin-1 expression in esophageal squamous cell carcinoma: a potent predictor of lymph node metastases, early tumor recurrence, and poor prognosis. Ann. Surg. Oncol. *21*, 329–336.

Jin, Y., Lee, S.J., Minshall, R.D., and Choi, A.M. (2011). Caveolin-1: a critical regulator of lung injury. Am. J. Physiol. Lung Cell. Mol. Physiol. *300*, L151–L160.

Joyce, J.A., and Pollard, J.W. (2009). Microenvironmental regulation of metastasis. Nat. Rev. Cancer *9*, 239–252.

Kaplan, R.N., Riba, R.D., Zacharoulis, S., Bramley, A.H., Vincent, L., Costa, C., MacDonald, D.D., Jin, D.K., Shido, K., Kerns, S.A., et al. (2005). VEGFR1-positive haematopoietic bone marrow progenitors initiate the pre-metastatic niche. Nature *438*, 820–827.

Koch, S., and Claesson-Welsh, L. (2012). Signal transduction by vascular endothelial growth factor receptors. Cold Spring Harb. Perspect. Med. *2*, a006502.

Koebel, C.M., Vermi, W., Swann, J.B., Zerafa, N., Rodig, S.J., Old, L.J., Smyth, M.J., and Schreiber, R.D. (2007). Adaptive immunity maintains occult cancer in an equilibrium state. Nature *450*, 903–907.

Kopf, M., Schneider, C., and Nobs, S.P. (2015). The development and function of lung-resident macrophages and dendritic cells. Nat. Immunol. *16*, 36–44.

Laoui, D., Van Overmeire, E., Di Conza, G., Aldeni, C., Keirsse, J., Morias, Y., Movahedi, K., Houbracken, I., Schouppe, E., Elkrim, Y., et al. (2014). Tumor hypoxia does not drive differentiation of tumor-associated macrophages but rather fine-tunes the M2-like macrophage population. Cancer Res. *74*, 24–30.

Lavin, Y., Mortha, A., Rahman, A., and Merad, M. (2015). Regulation of macrophage development and function in peripheral tissues. Nat. Rev. Immunol. *15*, 731–744.

Li, T., Zhu, Y., Han, L., Ren, W., Liu, H., and Qin, C. (2015). VEGFR-1 activation-induced MMP-9-dependent invasion in hepatocellular carcinoma. Future Oncol. *11*, 3143–3157.

Lin, E.Y., Jones, J.G., Li, P., Zhu, L., Whitney, K.D., Muller, W.J., and Pollard, J.W. (2003). Progression to malignancy in the polyoma middle T oncoprotein mouse breast cancer model provides a reliable model for human diseases. Am. J. Pathol. *163*, 2113–2126.

Lin, M.I., Yu, J., Murata, T., and Sessa, W.C. (2007). Caveolin-1-deficient mice have increased tumor microvascular permeability, angiogenesis, and growth. Cancer Res. *67*, 2849–2856.

López-Lago, M.A., Posner, S., Thodima, V.J., Molina, A.M., Motzer, R.J., and Chaganti, R.S. (2013). Neutrophil chemokines secreted by tumor cells mount a lung antimetastatic response during renal cell carcinoma progression. Oncogene *32*, 1752–1760.

Mantovani, A., and Allavena, P. (2015). The interaction of anticancer therapies with tumor-associated macrophages. J. Exp. Med. *212*, 435–445.

Moncho-Amor, V., Ibañez de Cáceres, I., Bandres, E., Martínez-Poveda, B., Orgaz, J.L., Sánchez-Pérez, I., Zazo, S., Rovira, A., Albanell, J., Jiménez, B., et al. (2011). DUSP1/MKP1 promotes angiogenesis, invasion and metastasis in non-small-cell lung cancer. Oncogene *30*, 668–678.

Morais, C., Ebrahem, Q., Anand-Apte, B., and Parat, M.O. (2012). Altered angiogenesis in caveolin-1 gene-deficient mice is restored by ablation of endothelial nitric oxide synthase. Am. J. Pathol. *180*, 1702–1714.

Müller-Hermelink, N., Braumüller, H., Pichler, B., Wieder, T., Mailhammer, R., Schaak, K., Ghoreschi, K., Yazdi, A., Haubner, R., Sander, C.A., et al. (2008). TNFR1 signaling and IFN-gamma signaling determine whether T cells induce tumor dormancy or promote multistage carcinogenesis. Cancer Cell *13*, 507–518.

Okuyama, H., Krishnamachary, B., Zhou, Y.F., Nagasawa, H., Bosch-Marce, M., and Semenza, G.L. (2006). Expression of vascular endothelial growth factor receptor 1 in bone marrow-derived mesenchymal cells is dependent on hypoxia-inducible factor 1. J. Biol. Chem. *281*, 15554–15563.

Ottewell, P.D., Coleman, R.E., and Holen, I. (2006). From genetic abnormality to metastases: murine models of breast cancer and their use in the development of anticancer therapies. Breast Cancer Res. Treat. *96*, 101–113.

Priceman, S.J., Sung, J.L., Shaposhnik, Z., Burton, J.B., Torres-Collado, A.X., Moughon, D.L., Johnson, M., Lusis, A.J., Cohen, D.A., Iruela-Arispe, M.L., and Wu, L. (2010). Targeting distinct tumor-infiltrating myeloid cells by inhibiting CSF-1 receptor: combating tumor evasion of antiangiogenic therapy. Blood *115*, 1461–1471.

Qian, B.Z., Li, J., Zhang, H., Kitamura, T., Zhang, J., Campion, L.R., Kaiser, E.A., Snyder, L.A., and Pollard, J.W. (2011a). CCL2 recruits inflammatory monocytes to facilitate breast-tumour metastasis. Nature *475*, 222–225.

Qian, N., Ueno, T., Kawaguchi-Sakita, N., Kawashima, M., Yoshida, N., Mikami, Y., Wakasa, T., Shintaku, M., Tsuyuki, S., Inamoto, T., and Toi, M. (2011b). Prognostic significance of tumor/stromal caveolin-1 expression in breast cancer patients. Cancer Sci. *102*, 1590–1596.

Qian, B.Z., Zhang, H., Li, J., He, T., Yeo, E.J., Soong, D.Y., Carragher, N.O., Munro, A., Chang, A., Bresnick, A.R., et al. (2015). FLT1 signaling in metastasis-associated macrophages activates an inflammatory signature that promotes breast cancer metastasis. J. Exp. Med. *212*, 1433–1448.

Ravid, D., Maor, S., Werner, H., and Liscovitch, M. (2005). Caveolin-1 inhibits cell detachment-induced p53 activation and anoikis by upregulation of insulin-like growth factor-I receptors and signaling. Oncogene *24*, 1338–1347.

Roda, J.M., Sumner, L.A., Evans, R., Phillips, G.S., Marsh, C.B., and Eubank, T.D. (2011). Hypoxia-inducible factor-2α regulates GM-CSF-derived soluble vascular endothelial growth factor receptor 1 production from macrophages and inhibits tumor growth and angiogenesis. J. Immunol. *187*, 1970–1976.

Ruffell, B., and Coussens, L.M. (2015). Macrophages and therapeutic resistance in cancer. Cancer Cell *27*, 462–472.

Simpkins, S.A., Hanby, A.M., Holliday, D.L., and Speirs, V. (2012). Clinical and functional significance of loss of caveolin-1 expression in breast cancer-associated fibroblasts. J. Pathol. *227*, 490–498.

Sosa, M.S., Bragado, P., and Aguirre-Ghiso, J.A. (2014). Mechanisms of disseminated cancer cell dormancy: an awakening field. Nat. Rev. Cancer *14*, 611–622.

Sunaga, N., Miyajima, K., Suzuki, M., Sato, M., White, M.A., Ramirez, R.D., Shay, J.W., Gazdar, A.F., and Minna, J.D. (2004). Different roles for caveolin-1 in the development of non-small cell lung cancer versus small cell lung cancer. Cancer Res. *64*, 4277–4285.

Tanase, C.P., Dima, S., Mihai, M., Raducan, E., Nicolescu, M.I., Albulescu, L., Voiculescu, B., Dumitrascu, T., Cruceru, L.M., Leabu, M., et al. (2009). Caveolin-1 overexpression correlates with tumour progression markers in pancreatic ductal adenocarcinoma. J. Mol. Histol. *40*, 23–29.

Williams, T.M., Medina, F., Badano, I., Hazan, R.B., Hutchinson, J., Muller, W.J., Chopra, N.G., Scherer, P.E., Pestell, R.G., and Lisanti, M.P. (2004). Caveolin-1 gene disruption promotes mammary tumorigenesis and dramatically enhances lung metastasis in vivo. Role of Cav-1 in cell invasiveness and matrix metalloproteinase (MMP-2/9) secretion. J. Biol. Chem. *279*, 51630–51646.

Witkiewicz, A.K., Dasgupta, A., Sammons, S., Er, O., Potoczek, M.B., Guiles, F., Sotgia, F., Brody, J.R., Mitchell, E.P., and Lisanti, M.P. (2010). Loss of stromal caveolin-1 expression predicts poor clinical outcome in triple negative and basal-like breast cancers. Cancer Biol. Ther. *10*, 135–143.

Wu, K.N., Queenan, M., Brody, J.R., Potoczek, M., Sotgia, F., Lisanti, M.P., and Witkiewicz, A.K. (2011). Loss of stromal caveolin-1 expression in malignant melanoma metastases predicts poor survival. Cell Cycle *10*, 4250–4255.

Yamaguchi, T., Murata, Y., Fujiyoshi, Y., and Doi, T. (2003). Regulated interaction of endothelin B receptor with caveolin-1. Eur. J. Biochem. *270*, 1816–1827.

Cell Reports, Volume *21*

Supplemental Information

Loss of Caveolin-1 in Metastasis-Associated Macrophages Drives Lung Metastatic Growth through Increased Angiogenesis

Ward Celus, Giusy Di Conza, Ana Isabel Oliveira, Manuel Ehling, Bruno M. Costa, Mathias Wenes, and Massimiliano Mazzone

SUPPLEMENTAL INFORMATION

	TUMOR-FREE MICE		TUMOR-BEARING MICE	
	WT→WT	Cav1 KO→WT	WT→WT	Cav1 KO→WT
WBC (K/μL)	12,40 ± 2,93	13,21 ± 3,57	67,25 ± 24,12	72,23 ± 24,01
NEU (%)	5,37 ± 0,95	5,92 ± 0,72	64,69 ± 12,22	69,70 ± 8,77
LYM (%)	87,43 ± 1,67	86,79 ± 2,53	15,50 ± 8,15	15,70 ± 6,61
MON (%)	3,09 ± 0,88	2,94 ± 0,71	3,02 ± 1,87	2,94 ± 1,63
EOS (%)	0,14 ± 0,05	0,21 ± 0,25	0,05 ± 0,04	0,06 ± 0,04
BAS (%)	3,71 ± 0,78	3,93 ± 0,12	3,12 ± 1,01	3,05 ± 0,04
RBC (M/μL)	8,90 ± 0,38	8,85 ± 0,49	4,23 ± 0,76	4,70 ± 0,98
PLT (K/μL)	175,50 ± 75,39	282,34 ± 116,71	180,26 ± 91,54	283,24 ± 78,84

Table S1. Hematological parameters of tumor-free and tumor-bearing WT→WT and Cav1 KO→WT chimeric mice. Related to Figure 1.

The values represent hematological parameters (mean ± SEM) in WT→WT and Cav1 KO→WT chimeric mice, measured before (tumor-free) and 14 days after the implantation of subcutaneous LLC tumors (tumor-bearing). Data are pooled from 2 independent experiments. Total mice, n = 10 per genotype.
Abbreviations: white blood cell (WBC), neutrophil (NEU), lymphocyte (LYM), monocyte (MON), eosinophil (EOS), basophil (BAS), red blood cell (RBC), platelet (PLT).

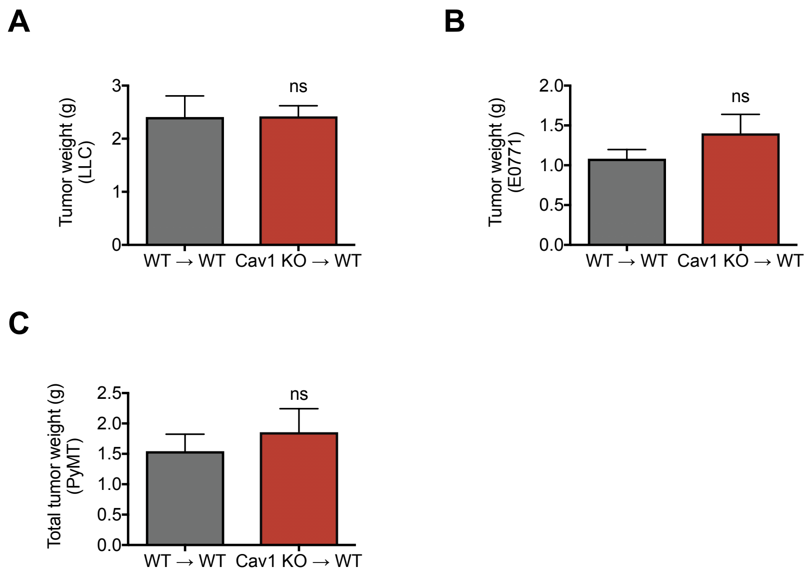

Figure S1. Cav1 deletion in the bone marrow does not affect primary tumor weight in multiple tumor models. Related to Figure 1.

(A) Tumor weight of WT→WT and Cav1 KO→WT chimeric mice in a subcutaneous LLC lung cancer model. Total mice, n = 30 per genotype.
(B) Tumor weight of WT→WT and Cav1 KO→WT chimeric mice in an orthotopic E0771 breast cancer model. Total mice, n = 8 per genotype.
(C) Total tumor weight of lethally irradiated PyMT mice reconstituted with WT or Cav1 KO bone marrow cells. Total mice, n = 7 per genotype.
All graphs show mean ± standard error of the mean (SEM). ns, not significant.

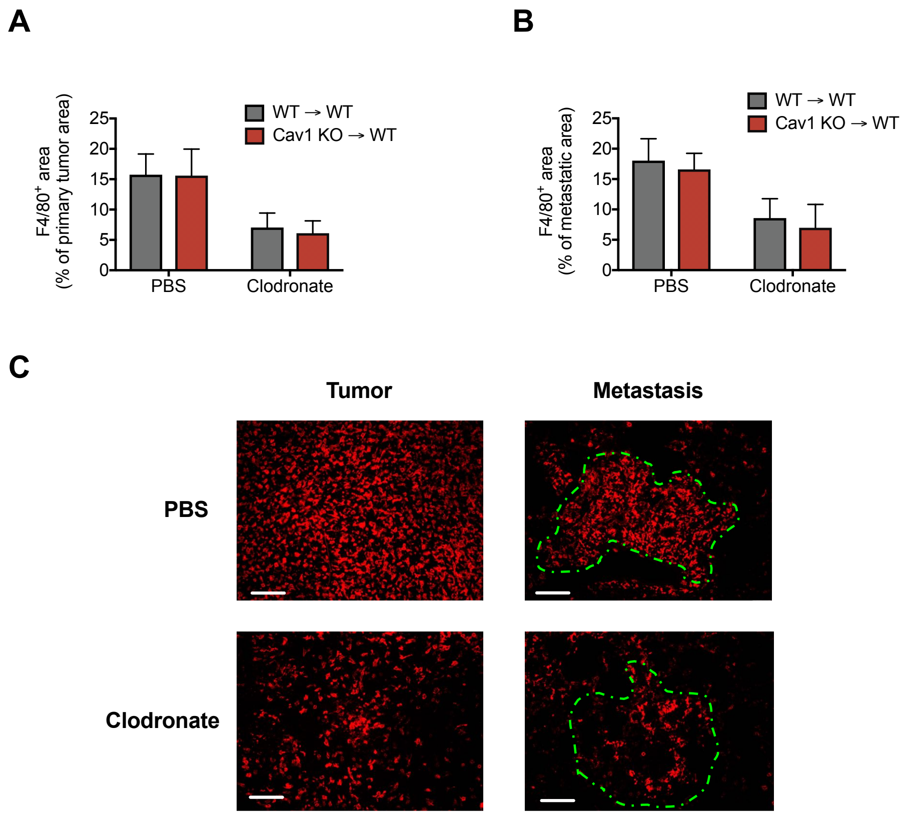

Figure S2. Macrophage infiltration is unaffected in both primary tumor and metastasis upon Cav1 deletion in the bone marrow. Related to Figure 2.

(A-C) Percentage of F4/80$^+$ macrophages upon treatment with macrophage-depleting clodronate liposomes or PBS in LLC primary tumors (A) and LLC metastatic lung lesions (B). Representative pictures of clodronate efficacy in both primary tumor and metastasis (C). Scale bar: 100 µm. Total mice, n = 10 per genotype.
All graphs show mean ± SEM.

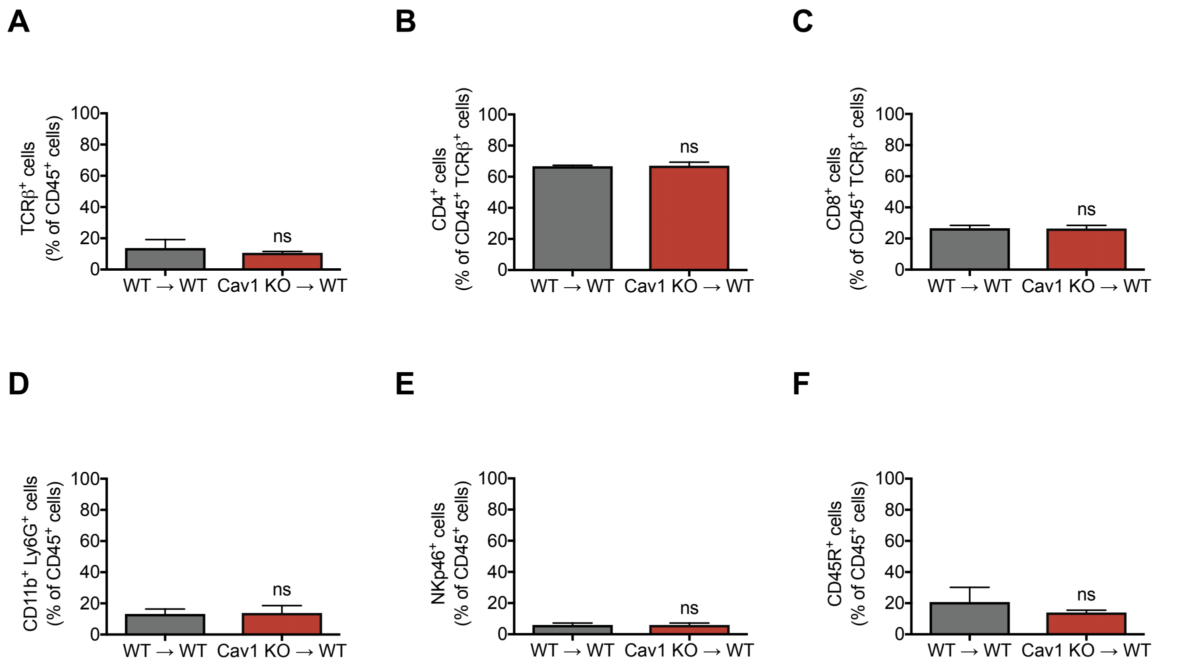

Figure S3. Cav1 deletion in the bone marrow does not affect immune cell infiltration in the metastasis. Related to Figure 4.

(A-F) FACS quantification of total TCRβ$^+$ cells (A), CD4$^+$ T cells (B), CD8$^+$ T cells (C), CD11b$^+$Ly6G$^+$ neutrophils (D), NKp46$^+$ NK cells (E) and CD45R$^+$ B cells (F) in WT and Cav1 KO lung metastatic lesions. Total mice, n = 4 per genotype.
All graphs show mean ± SEM. ns, not significant.

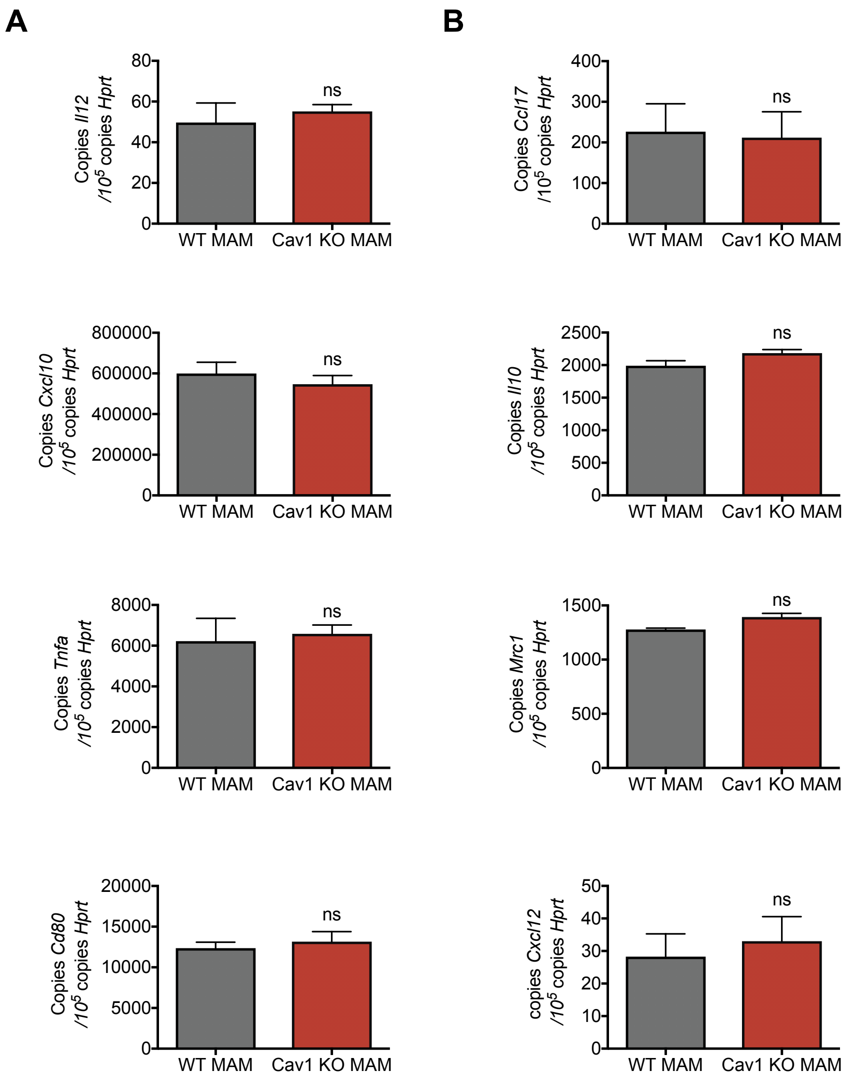

Figure S4. M1-like and M2-like markers are unaffected upon Cav1 deletion in sorted MAMs. Related to Figure 4.

(A and B) qRT-PCR quantification of M1-like markers IL-12, CXCL10, TNFα and CD80 (C) and M2-like markers CCL17, IL-10, Mrc1 and CXCL12 in WT and Cav1 KO sorted MAMs from lung metastatic lesions. Total mice, n = 4 per genotype.
All graphs show mean ± SEM. ns, not significant.

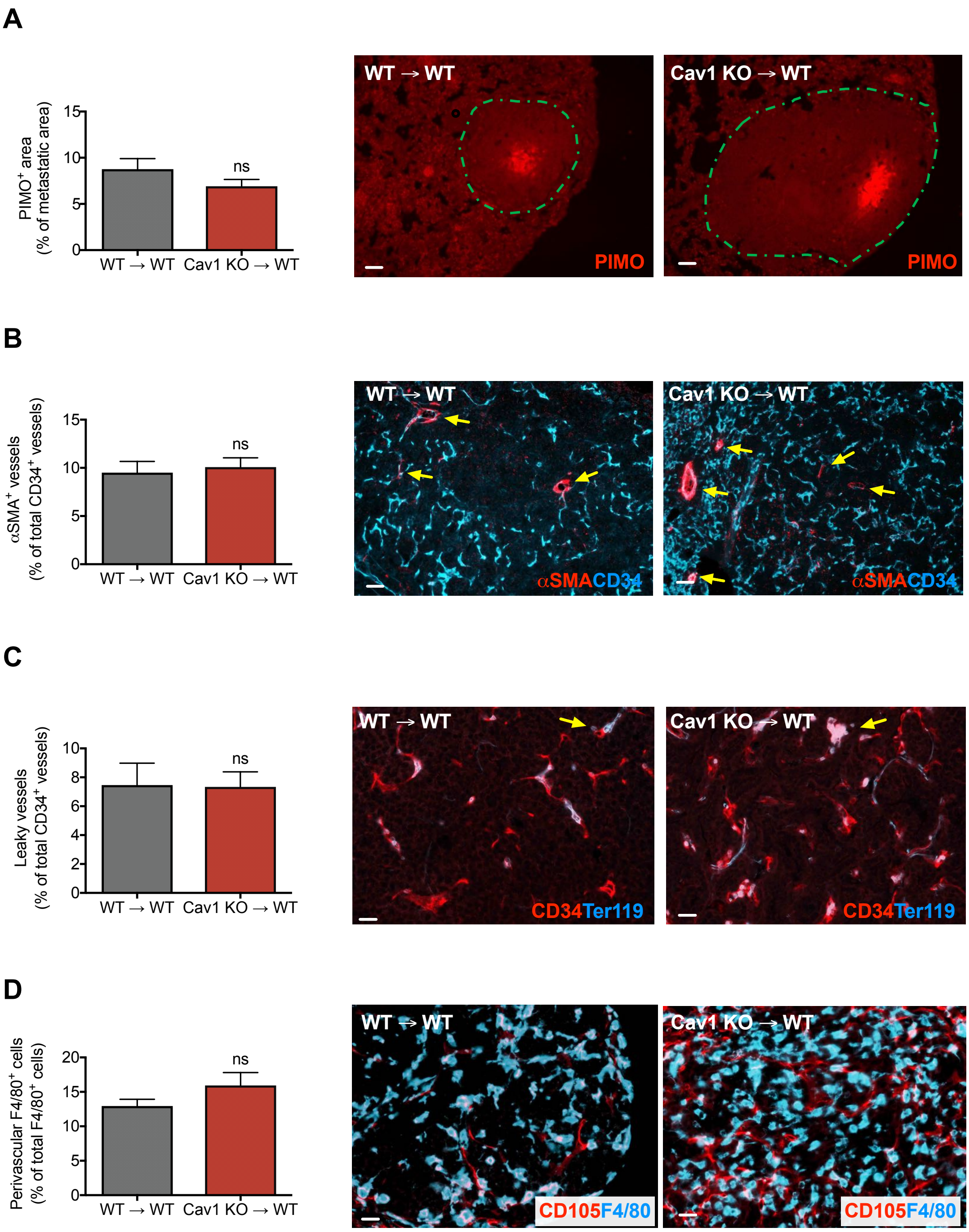

Figure S5. The functionality and vascular integrity of blood vessels in the pulmonary metastases are unchanged upon Cav1 deletion in BMDCs. Related to Figure 4.

(A) Quantification and representative images of $PIMO^+$ (pimonidazole) hypoxic areas in WT and Cav1 KO lung metastatic lesions. Scale bar: 100 µm. Total mice, n = 7 per genotype.
(B) Quantification and representative images of αSMA^+ pericyte-covered vessels (arrows) over the total number of CD34+ vessels in WT and Cav1 KO lung metastatic lesions. Scale bar: 50 µm. Total mice, n = 7 per genotype.
(C) Quantification and representative images of leaky vessels in WT and Cav1 KO lung metastatic lesions, measured as the percentage of $CD105^+$ vessels surrounded by $Ter119^+$ red blood cells (arrows) over the total number of vessels. Scale bar: 50 µm. Total mice, n = 7 per genotype.
(D) Quantification and representative pictures of perivascular macrophages measured as the percentage of perivascular $F4/80^+$ macrophages over the total number of $F4/80^+$ macrophages in the metastatic lesion. Scale bar: 50 µm. Total mice, n = 4 per genotype.
All graphs show mean ± SEM. ns, not significant.

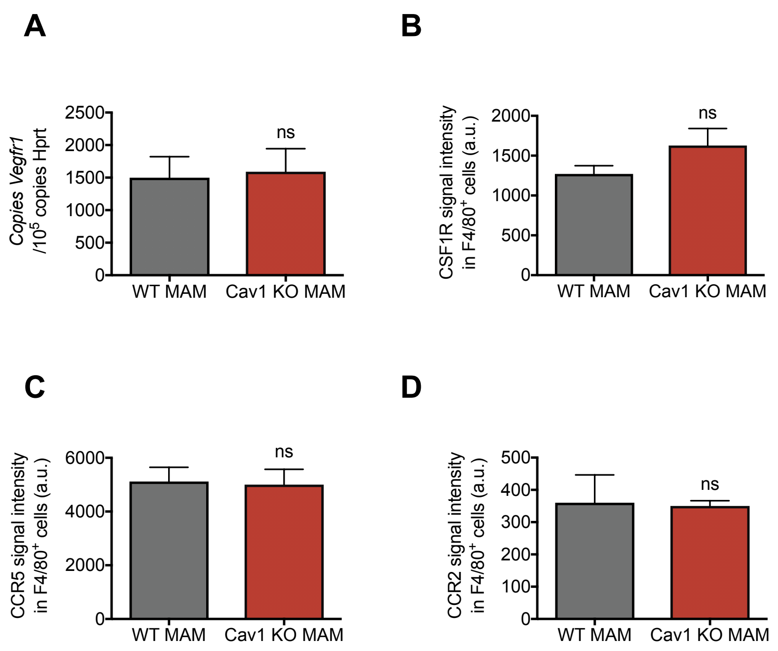

Figure S6. Cav1 deletion in MAMs leads to a specific membrane deficiency in VEGFR1. Related to Figure 5.

(A) *Vegfr1* expression in sorted $CD45^+CD11b^+F4/80^+$ MAMs. Total mice, n = 6 per genotype.
(B-D) FACS analysis of the mean CCR2, CSF-1R and CCR5 signal intensity on $CD45^+CD11b^+F4/80^+$ macrophages in lung metastatic lesions. Total mice, n = 6 per genotype.
All graphs show mean ± SEM. ns, not significant.

SUPPLEMENTAL EXPERIMENTAL PROCEDURES

Animals

Cav1 KO mice on a C57BL/6 background were obtained from Dr. Feron (UCLouvain, Brussels, Belgium). C57BL/6 mice were purchased from Charles River. All mice used were between 5 and 13 weeks old, without specific gender selection. In all experiments, littermate controls were used. Housing and all experimental animal procedures were approved by the Institutional Animal Care and Research Advisory Committee of the KU Leuven.

Bone marrow transplantation

6-week-old C56BL/6 recipient mice were lethally irradiated with 9.5 Gy. Subsequently, 10 x 10^6 bone marrow cells from the appropriate genotype were injected intravenously via tail vein. Tumor experiments were initiated 6 to 8 weeks after bone marrow reconstitution. Red and white blood cell count was determined using a hemocytometer on peripheral blood, collected in heparin with capillary pipettes by retro-orbital bleeding.

Cell lines

Murine Lewis lung carcinoma cells (LLC) and the E0771 medullary breast adenocarcinoma cells were obtained from the American Type Culture Collection (ATCC) and CH3Biosystems, respectively. The cells were cultured in DMEM (Gibco) supplemented with 10% Fetal Bovine Serum (FBS, Gibco), 2 mM glutamine, 100 units/ml penicillin and 100 µg/ml streptomycin. All cells were passaged in the laboratory for no longer than 6 months after receipt.

Tumor models

1 x 10^6 LLC adherent growing murine cells were injected subcutaneously at the right side of the mouse in a volume of 200 µl of PBS. Alternatively, 5 x 10^5 E0771 medullary breast adenocarcinoma cells were injected orthotopically in the mammary fat pad of the second nipple on the right side in a volume of 50 µl of PBS. Tumor volumes were measured three times a week with a caliper and calculated using the formula: $V = \pi \times d^2 \times D/6$, where d is the minor tumor axis and D is the major tumor axis. At the end stage, tumor weight was measured and lung metastasis nodules were contrasted after intratracheal injection of 15% India ink solution or by H&E staining on lung paraffin sections. Superficial metastatic nodules were assessed under a stereomicroscope. MMTV-PyMT spontaneous breast tumors were measured 20 weeks after birth (8 weeks after bone marrow transplantation), twice a week with a caliper, and mice were killed at week 25.

Macrophage depletion

Macrophage depletion was achieved by intraperitoneal injection of a loading dose of 250 µl clodronate and control PBS liposomes, purchased in suspension from ClodLip (ClodronateLiposomes, The Netherlands) (Van Rooijen and Sanders, 1994). 12h later, LLC tumor cells were injected subcutaneously followed by another dose of 250 µl liposomes 6h after injection. During tumor progression, repeated injections of 250 µl every second day to prevent repopulation of macrophages. The efficiency of macrophage depletion was assessed by immunostainings of tumor and lung sections for F4/80.

LLC lung extravasation

To determine the kinetics of tumor cell extravasation in the lung, we transduced LLC cells with a lentivirus expressing a puromycin resistance gene. After puromycin selection, 5 x 10^5 LLC cells in 200 µl PBS were injected directly in the bloodstream. Lungs were dissected at the indicated time points and snap frozen in liquid nitrogen. Whole lungs were homogenized by a ribolyser (MPBio) and gDNA was isolated by the DNeasy Blood and Tissue Kit (Qiagen). The number of LLC cells present in the lungs was measured by qPCR on 2 µg gDNA by using primers against the lentiviral backbone, normalizing with a standard curve containing 2-fold dilutions of the transduced LLC cells.

LLC lung colonization experiments

5 x 10^5 LLC cells in 200 µl PBS were injected directly in the bloodstream. VEGFR1 inhibition was achieved by i.p. injection of 20 mg/kg mouse anti-VEGFR1 antibodies (clone MF1, Thrombogenics) or isotype IgG control (Sigma-Aldrich) every second day. MMP9 inhibition was achieved by daily gavage injection of 15 mg/kg MMP9-inibitor II (444293 from Millipore), previously diluted in 50% methylcellulose. DC101 treatment was achieved by i.p. injection of 40 mg/kg rat anti-mouse VEGFR2 antibodies (BioXcell) twice a week. Blockage of VEGF-A was achieved by i.p. injection of 40 mg/kg chimeric anti-mouse VEGF-A (clone B20, ThromboGenics) or isotype IgG control (Sigma-Aldrich). Anti-VEGF injections were performed at day 16 and 18 upon LLC tail vein injection. For all treatments, mice were dissected after 20 days and lung metastatic nodules were contrasted after intratracheal injection of 15% India ink solution.

Portal vein injection of E0771 breast cancer cells

For intraportal tumor cell injection, laparotomy was performed through a midline incision. Exponentially growing E0771 cultures were trypsinized, washed and resuspended at a concentration of 1×10^5 cells in 100 µl of PBS. The cells were injected in the portal vein using a 30-gauge syringe. Hemorrhagic control was performed using absorbable collagen swabs for compression at the injection site. After injection, the abdominal wall was closed by a suture and animals were allowed to recover on a warming pad in a separate cage until completely conscious. Mice were killed at day 10 and liver metastatic nodules were contrasted by H&E staining on liver paraffin sections (Goddard et al., 2016).

Histology and immunostainings

Serial sections were cut at 7 µm thickness and tissue samples were fixed in 2% PFA overnight at 4°C, dehydrated and embedded in paraffin. Paraffin slides were first rehydrated to further proceed with antigen retrieval in citrate solution (DAKO). If necessary, 0.3% hydrogen peroxide was added to methanol, to block endogenous peroxidases. The sections were blocked with the appropriate serum (DAKO) and incubated overnight with the following antibodies: rat anti-CD31 (BD Pharmingen), rat anti-F4/80 (Serotec), anti-SMA-Cy3 (Sigma Aldrich), rabbit anti-hypoxyprobe (HPI), goat anti-CD105, rat anti-Ter119 (R&D systems) and Hoechst (Thermo Fisher Scientific). Appropriate secondary antibodies were used: Alexa 488, 647 or 568 conjugated secondary antibodies (Molecular Probes), biotin-labeled antibodies (Jackson Immunoresearch) and, when necessary, Tyramide/DAB (Sigma-Aldrich) or TSA Plus Cyanine 3 and Cyanine 5 System amplification (Perkin Elmer, Life Sciences) were performed according to the manufacturer's instructions. Whenever sections were stained in fluorescence, ProLong Gold mounting medium with or without DAPI (Invitrogen) was used. Microscopic analysis was done with an Olympus BX41 microscope and CellSense imaging software. Macrophage and vessel analysis was performed as previously described (Palmieri et al., 2017; Wenes et al., 2016).

Hypoxia assessment

Tumor hypoxia was detected 1h after i.p. injection of 60 mg/kg pimonidazole hydrochloride into tumor-bearing mice. Mice were sacrificed and metastases were harvested. To detect the formation of pimonidazole adducts, metastasis paraffin sections were immunostained with Hypoxyprobe-1-Mab1 (Hypoxyprobe kit, Chemicon) following the manufacturer's instructions.

FACS analysis and flow sorting of tumor- and metastasis-associated macrophages

LLC tumor-bearing mice were sacrificed by cervical dislocation and tumors and macroscopic lung metastasis were harvested. Tumors and metastases were minced in RPMI medium containing 0.1% collagenase type I and 0,2% dispase type I and incubated in the same solution for 30 minutes at 37°C. The digested tissue was filtered using a 70 µm pore sized mesh and cells were centrifuged 5 min at 1000 rpm. Red blood cell lysis was performed by using Hybri-Max^{TM} (Sigma-Aldrich). Cells were resuspended in FACS buffer (PBS containing 2% FBS and 2 mM EDTA) and incubated for 15 minutes with Mouse BD Fc Block purified anti-mouse CD16/CD32 mAb (BD-Pharmingen) and stained with the following antibodies for 30 minutes at 4 °C: Fixable viability dye, anti-CD11b, anti-F4/80, anti-CD11C, anti-CD4, anti-CD8, anti-MHCII, anti-CD206 (Mrc1) (All from Thermo Fisher Scientific), anti-CD45, anti-TCRβ, anti-CD45R (BD Biosciences), anti-FLT1 (R&D Systems), anti-Ly6G, anti-CD335 (NKp46), anti-CCR2, anti-CCR5 and anti-CSF-1R (All from Biolegend). Cells were subsequently washed and resuspended in FACS buffer before FACS analysis or flow sorting by a FACS Verse or FACS Aria III (BD Biosciences), respectively.

Blood and spleen-derived monocytes

For the collection of monocytes, spleen samples were mechanically dissociated, filtered using a 70 µm pore sized mesh and the cells were centrifuged 5 min at 1000 rpm. Blood samples were collected in heparin with capillary pipettes by retro-orbital bleeding. Red blood cell lysis was performed by using Hybri-MaxTM (Sigma-Aldrich). The cells were resuspended in FACS buffer (PBS containing 2% FBS and 2 mM EDTA) and incubated for 15 minutes with Mouse BD Fc Block purified anti-mouse CD16/CD32 mAb (BD-pharmingen) and stained with the following antibodies for 30 minutes at 4 °C: Fixable viability dye, anti-F4/80 (Thermo Fisher Scientific), anti-Ly6C (BD Biosciences) and anti-Ly6G (Biolegend). Cells were subsequently washed and resuspended in cold FACS buffer before flow sorting by a FACS Aria (BD Biosciences). Afterwards, the monocytes were treated for 7 days in culture with 50 ng/ml GM-CSF (Biolegend).

Bone marrow-derived macrophages (BMDMs)

Murine bone marrow-derived macrophages (BMDMs) were derived from bone marrow precursors as described before (Meerpohl et al., 1976). Briefly, bone marrow cells (1,6 x10^6 cells/ml) were cultured in a volume of 6 ml in a 10 cm Petri dish (non-tissue culture treated, bacterial grade) in DMEM supplemented with 20% FBS and 30%

L929 conditioned medium as a source of M-CSF. After 3 days of culture, an additional 3 ml of differentiation medium was added. At day 7, the macrophages were considered differentiated and lysed with RLT buffer (Qiagen) for RNA extraction.

ELISA detection of secreted VEGF-A

The VEGF-A levels in the cell culture medium of sorted MAMs were assessed by ELISA. Following incubation of 2 days, the culture supernatant was collected and VEGF-A levels were measured using a mouse VEGF ELISA kit (Invitrogen, KMG0111) according to the manufacturer's instructions. The absorbance at 450 nm was determined using a microplate reader.

Quantitative RT-PCR

Quantitative RT-PCR was performed as previously described (Fischer et al., 2007), using commercially available (Applied Biosystems and IDT) or homemade primers for the studied genes. The sequence of the primers are available upon request.

MMP9 assay

The gelatinolytic activity of MMP9 was tested using a SDS-page gelatin zymography. Briefly, enzymes were separated based on their molecular weight on 7,5% polyacrylamide-SDS gels containing 1 mg/ml porcine gelatin (Sigma), under non-reducing conditions. For the zymography, a Mini-PROTEAN® III system (Bio-Rad) was used. Protein concentrations of the supernatant from FACS sorted MAMs were determined using a bicinchoninic acid (BCA) Protein Assay Kit (Pierce) according to the manufacturer's instructions. Molecular weight of the gelatinolytic areas was estimated by using Precision Plus Protein Dual Color Standards (Bio-Rad). After migration, gels were washed twice with 2.5% Triton-X100 at room temperature (RT) for 30 min, and incubated with an activation buffer (50 mM Tris-HCl, 10 mM $CaCl_2$, pH 7.5) overnight at 37 °C. Gels were then stained with 0,5% Coomassie Brilliant Blue-R250 for 30 min and destained with a destain buffer (30% methanol, 10% acetic acid) for 60 min at RT. The gelatinolytic activity was visualized as clear bands corresponding to the digested areas. A brighter or a larger band indicated a more concentrated proteinase in the sample. Relative MMP9 activity was analyzed by densitometry using ImageJ.

Statistics

Data entry and all analyses were performed in a blinded fashion. All statistical analyses were performed using GraphPad Prism software on mean values, calculated from the averages of technical replicates. Statistical significance was calculated by two-tailed unpaired t-test on two experimental conditions or two-way ANOVA when repeated measures were compared, with $p < 0.05$ considered statistically significant. Data were tested for normality using the D'Agostino–Pearson omnibus test (for $n > 8$) or the Kolmogorov–Smirnov test (for $n \leq 8$) and variation within each experimental group was assessed. Detection of mathematical outliers was performed using the Grubbs' test in GraphPad. Sample sizes for all experiments were chosen based on previous experiences. Independent experiments were pooled and analyzed together whenever possible. All graphs show mean values ± SEM.

SUPPLEMENTAL REFERENCES

Fischer, C., Jonckx, B., Mazzone, M., Zacchigna, S., Loges, S., Pattarini, L., Chorianopoulos, E., Liesenborghs, L., Koch, M., De Mol, M., *et al.* (2007). Anti-PlGF inhibits growth of VEGF(R)-inhibitor-resistant tumors without affecting healthy vessels. Cell *131*, 463-475.

Goddard, E.T., Fischer, J., and Schedin, P. (2016). A Portal Vein Injection Model to Study Liver Metastasis of Breast Cancer. J Vis Exp.

Meerpohl, H.G., Lohmann-Matthes, M.L., and Fischer, H. (1976). Studies on the activation of mouse bone marrow-derived macrophages by the macrophage cytotoxicity factor (MCF). Eur J Immunol *6*, 213-217.

Palmieri, E.M., Menga, A., Martín-Pérez, R., Quinto, A., Riera-Domingo, C., De Tullio, G., Hooper, D.C., Lamers, W.H., Ghesquière, B., McVicar, D.W., *et al.* (2017). Pharmacologic or Genetic Targeting of Glutamine Synthetase Skews Macrophages toward an M1-like Phenotype and Inhibits Tumor Metastasis. Cell Rep *20*, 1654-1666.

Van Rooijen, N., and Sanders, A. (1994). Liposome mediated depletion of macrophages: mechanism of action, preparation of liposomes and applications. Journal of immunological methods *174*, 83-93.

Wenes, M., Shang, M., Di Matteo, M., Goveia, J., Martín-Pérez, R., Serneels, J., Prenen, H., Ghesquière, B., Carmeliet, P., and Mazzone, M. (2016). Macrophage Metabolism Controls Tumor Blood Vessel Morphogenesis and Metastasis. Cell Metab *24*, 701-715.
